# *N*-Sulfenylsuccinimide/phthalimide: an alternative sulfenylating reagent in organic transformations

**DOI:** 10.3762/bjoc.19.106

**Published:** 2023-09-27

**Authors:** Fatemeh Doraghi, Seyedeh Pegah Aledavoud, Mehdi Ghanbarlou, Bagher Larijani, Mohammad Mahdavi

**Affiliations:** 1 Endocrinology and Metabolism Research Center, Endocrinology and Metabolism Clinical Sciences Institute, Tehran University of Medical Sciences, Tehran, Iran

**Keywords:** electrophile, *N*-(sulfenyl)succinimides/phthalimides, organic transformations, organosulfur, sulfenylation

## Abstract

In the field of organosulfur chemistry, sulfenylating agents are an important key in C–S bond formation strategies. Among various organosulfur precursors, *N*-sulfenylsuccinimide/phthalimide derivatives have shown highly electrophilic reactivity for the asymmetric synthesis of many organic compounds. Hence, in this review article, we focus on the application of these alternative sulfenylating reagents in organic transformations.

## Introduction

**Scheme 1:**
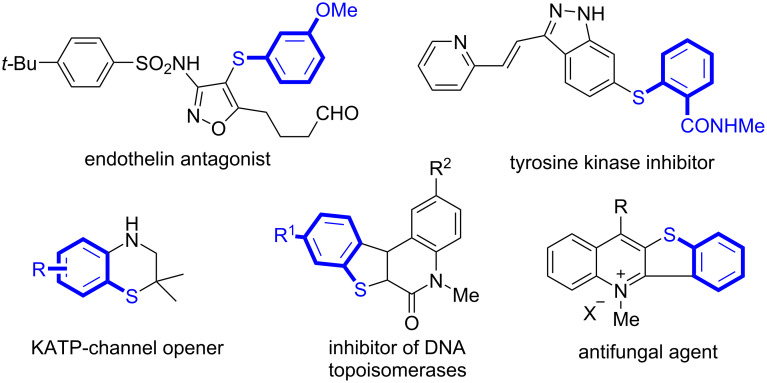
Sulfur-containing bioactive molecules.

Sulfur-containing compounds are of high importance in organic synthesis, medicinal chemistry, and materials science [1–5]. For example, they are used in the treatment of cancer [6–8], inflammation [9–11], human immunodeficiency virus [12–13], Alzheimer’s and Parkinson’s diseases [14–15]. Scheme 1 shows selected examples of sulfur-containing pharmaceutical molecules. Considering the synthetic applications of sulfur-based compounds, a large number of researchers have noted that these scaffolds have promising potential for the research and development of new biomedicines.

In the sulfenylation of organic compounds, the sulfenylating agents are important factors, and the commonly utilized chemicals include thiols [16–18], disulfides [19–22], sulfenyl halides [23–25], sulfonamides [26], sulfenate esters [27–28], and methyl(bismethylthio)sulfonium salts [29–30]. Among various organic molecules, aryl sulfides are recognized as functional materials and indispensable synthetic intermediates in drug discovery [31–33]. Because of their value, constructing C–S bonds has attracted significant attention via metal-catalyzed cross-coupling reactions and metal-free C–S bond formation [34–37]. Direct sulfenylation of the C–H bonds of unactivated aryls or aromatic sulfenylation by electrophilic aromatic substitution (S_E_Ar) has also recently received attention [38].

In recent years, *N*-(aryl/alkylsulfenyl)succinimides and *N*-(arylsulfenyl)phthalimides have been widely employed as new alternative sulfenylating reagents in the field of organic synthesis. These compounds are readily accessible, safe, and more stable than toxic, unstable, and foul-smelling thiols. These electrophilic sulfur sources have deserved particular interest for the C–S bond formation via the reaction with various nucleophiles. Their preparation is usually a two-step procedure, involving a treatment of the thiol with sulfuryl chloride in the presence of Et_3_N and the addition of the resulting solution to a mixture of succinimide/phthalimide and Et_3_N in the next step [39–40].

According to the irreplaceable role of sulfur-based frameworks in materials science and the pharmaceutical area, there is a force for researchers to identify sustainable methodologies for efficient C–S bond coupling under mild reaction conditions for achieving these distinguished compounds. Recently, several reviews about sulfenylating reagents have been reported [41–43]. To the best of our knowledge there are no review articles focusing on the application of *N*-(sulfenyl)succinimides/phthalimides in sulfenylation reactions. In this context, we describe various sulfenylation reactions, such as electrophilic aromatic substitution, ring-opening, dehydrogenative cross-coupling, and direct sulfenylation reactions, which are classified into three categories: sulfenylation catalyzed by i) transition metal catalysts, ii) organocompound catalysts, and iii) catalyst-free sulfenylation.

## Review

### Sulfenylation of organic compounds by *N*-(sulfenyl)succinimides/phthalimides

#### Metal-catalyzed sulfenylation by *N*-(sulfenyl)succinimides/phthalimides

**Scheme 2:**
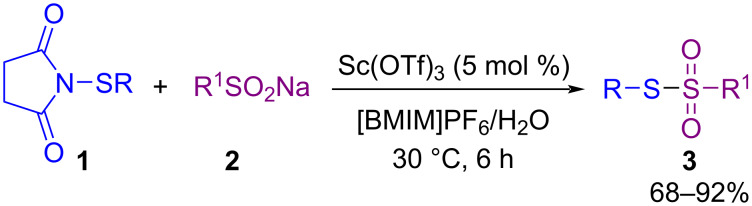
Scandium-catalyzed synthesis of thiosulfonates.

In 2012, Chen and co-workers found that in the reaction of *N*-(organothio)succinimides **1** and sodium sulfinates **2** using a Lewis acid in ionic liquids (ILs) and water as a green solvent system leads to the formation of thiosulfonates **3** (Scheme 2) [44]. Among different Lewis acid catalysts, such as Cu(OTf)_2_, Mg(OTf)_2_, Zn(OTf)_2_, Sc(OTf)_3_, Eu(OTf)_3_, and Yb(OTf)_3_, it was found that Sc(OTf)_3_ gave higher product yield. In addition, the combination of Sc(OTf)_3_/ILs displayed good recyclability in this transformation.

**Scheme 3:**
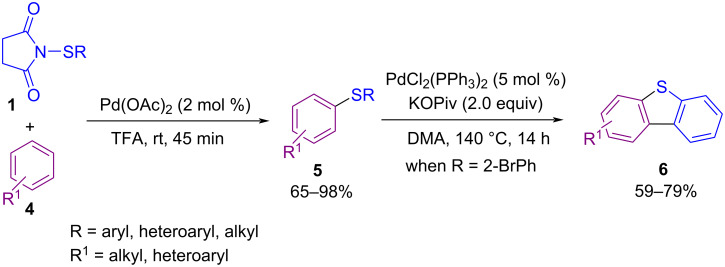
Palladium-catalyzed aryl(alkyl)thiolation of unactivated arenes.

**Scheme 4:**
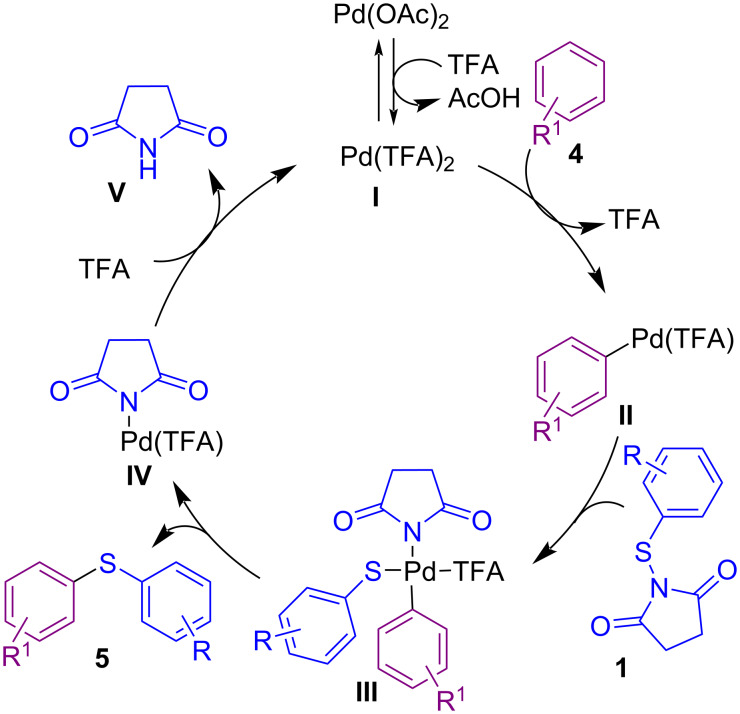
Catalytic cycle for Pd-catalyzed aryl(alkyl)thiolation of unactivated arenes.

In 2014, Anbarasan and Saravanan succeeded in synthesizing various diaryl(alkyl) sulfides **5** through the sulfenylation of unactivated arenes **4** with an electrophilic sulfur reagent in the presence of a palladium catalyst (Scheme 3) [45]. In the second phase, dibenzothiophene derivatives **6** were obtained via subsequent intramolecular arylation of aryl sulfides by using the catalyst and the base. A catalytic cycle is shown in Scheme 4. Firstly, electrophilic Pd(TFA)_2_ generated from Pd(OAc)_2_ and TFA, which (by C–H functionalization of arene **4**) led to intermediate **II**. Oxidative insertion of intermediate **II** into the N–S bond of **1** afforded intermediate **III**. Reductive elimination of Pd from **III** gave product **5** and species **IV**. Finaly, Pd(II) species were reproduced by ligand exchange to restart the next cycle (Scheme 4).

**Scheme 5:**
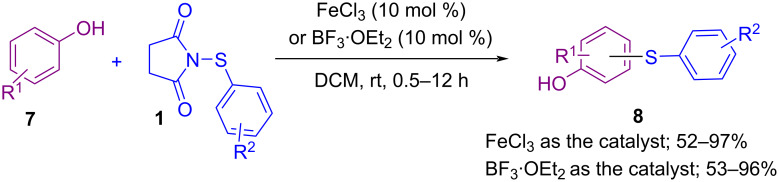
Iron- or boron-catalyzed C–H arylthiation of substituted phenols.

In 2014, Fu and co-workers described a facile method for the C–H thiolation of phenols **7** with 1-(substituted phenylthio)pyrrolidine-2,5-diones **1** using FeCl_3_ or BF_3_·OEt_2_ as a catalyst (Scheme 5) [46]. A wide variety of thiolated phenols **8** were produced under mild reaction conditions without using any base, ligand, or additive. For both substrates, **7** and **1** aryl rings containing electron-donating groups exhibited a higher reactivity than electron-withdrawing groups, and the thiolation occurred mainly at the *para* position to the hydroxy group in phenols.

**Scheme 6:**
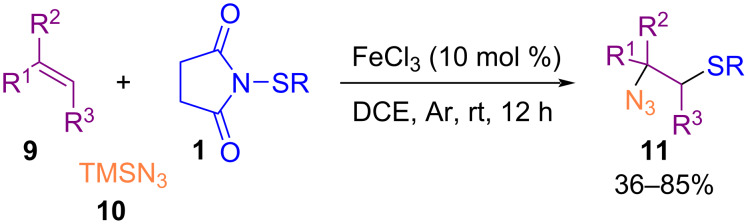
Iron-catalyzed azidoalkylthiation of alkenes.

**Scheme 7:**
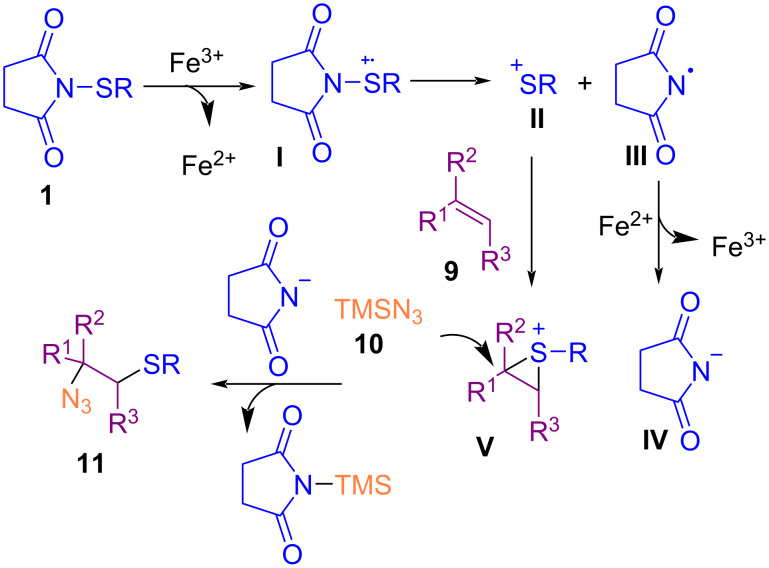
Plausible mechanism for iron-catalyzed azidoalkylthiation of alkenes.

In 2016, the azidoarylthiation of various alkenes **9** by trimethylsilyl azide (**10**) and *N*-(organothio)succinimide **1** to the corresponding products containing *ortho*-sited azide and sulfide moieties **11** was performed by Fu et al. (Scheme 6) [47]. Iron(III) chloride was used as a catalyst for this coupling reaction without the need of any ligand and additive. Screening for other metal salts, such as Cu(OAc)_2_, Pd(OAc)_2_, AgOAc or CuI was not successful, although FeS·7H_2_O, FeS, Fe_2_(SO_4_)_3_·H_2_O, FeSO_4_, and Fe(acac)_3_ resulted in inferior chemical yields. Employment of 2,2,6,6-tetramethylpiperidinyl-1-oxyl (TEMPO) as a radical trapper inhibited the reaction, which proved that a radical process was involved. The reaction was initiated by a single electron transfer (SET) process from the sulfur atom to Fe^3+^ to generate Fe^2+^ and radical cation **I**. Subsequent cleavage of the N–S bond led to cation **II** and radical **III**. Interaction of **III** with Fe^2+^ regenerated the Fe^3+^ species and **IV**. At the same time, electrophilic addition of **II** to alkene **9** yielded intermediate **V**, which was subjected to the nucleophilic attack of TMSN_3_ to deliver product **11** (Scheme 7).

**Scheme 8:**
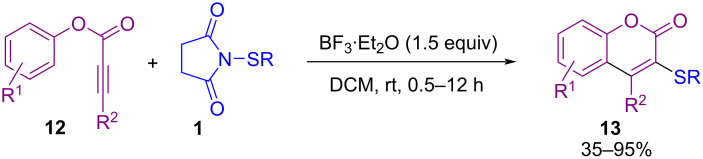
BF_3_·Et_2_O‑mediated electrophilic cyclization of aryl alkynoates.

**Scheme 9:**
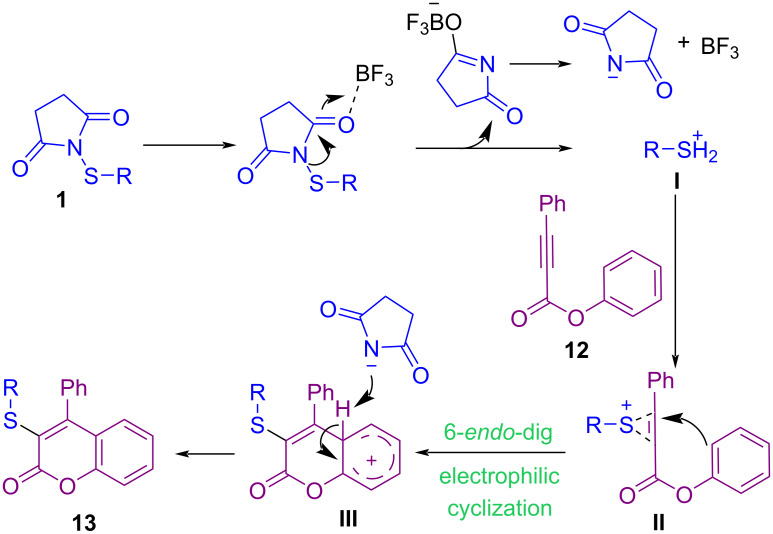
Tentative mechanism for BF_3_·Et_2_O‑mediated electrophilic cyclization of aryl alkynoates.

Tian and Chang et al. could synthesize 3‑sulfenylated coumarin compounds **13** by using *N*-sulfanylsuccinimides **1** under a Lewis acid catalysis system (Scheme 8) [48]. Additionally, oxidation of 3-sulfenylated coumarins utilizing (diacetoxyiodo)benzene (PIDA) and *meta*-chloroperbenzoic acid (*m*-CPBA) toward 3-sulfinylated and 3-sulfonylated product, respectively, were performed in this work. A plausible mechanism involves the treatment of **1** with BF_3_·Et_2_O toward cation **I**, which reacted with the C–C triple bond in **12** to give sulfonium intermediate **II**. Intramolecular nucleophilic addition of the phenoxy ring of **12** to the activated C–C triple bond afforded intermediate **III**, followed by deprotonation to deliver product **13** (Scheme 9). When substrate **12** had an OMe group on the phenoxy ring, ipso sulfenylcyclization, or sulfenylation of the phenoxy ring occurred according to the different positions of the OMe group. The preparation of α,α-bisthiofunctionalized butenolides through a bis-sulfenylation methodology was reported by Zhou and Yuan et al. [49]. For this purpose, they applied *N*-(alkyl(aryl)sulfanyl)succinimides or *N*-(phenylsulfanyl)phthalimides using a catalytic amount of Et_3_N. Moreover, mono-sulfenylation of α-methyl-γ-phenyl-substituted butenolide at α-position was carried out in the presence of Et_3_N as well as quinine organocatalyst and products were obtained in high yields.

**Scheme 10:**
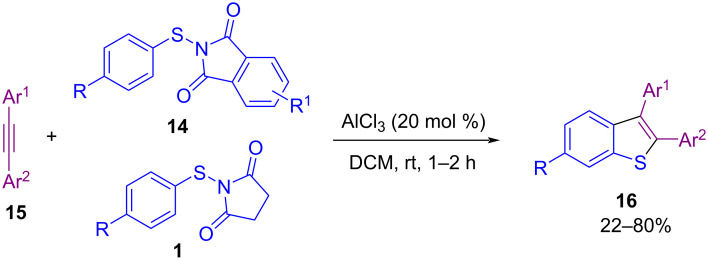
Construction of 6-substituted benzo[*b*]thiophenes.

**Scheme 11:**
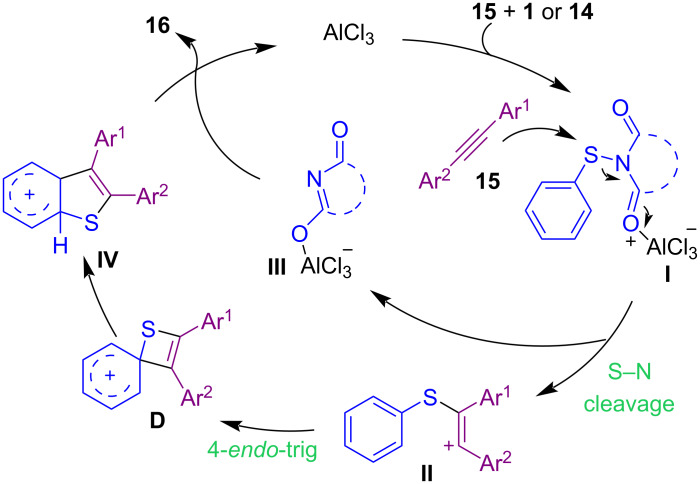
Plausible mechanism for construction of 6-substituted benzo[*b*]thiophenes.

**Scheme 12:**
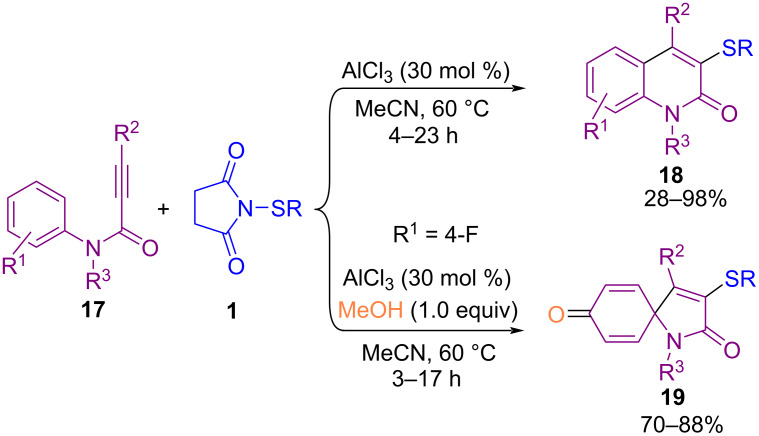
AlCl_3_‑catalyzed cyclization of *N*‑arylpropynamides with *N*‑sulfanylsuccinimides.

**Scheme 13:**
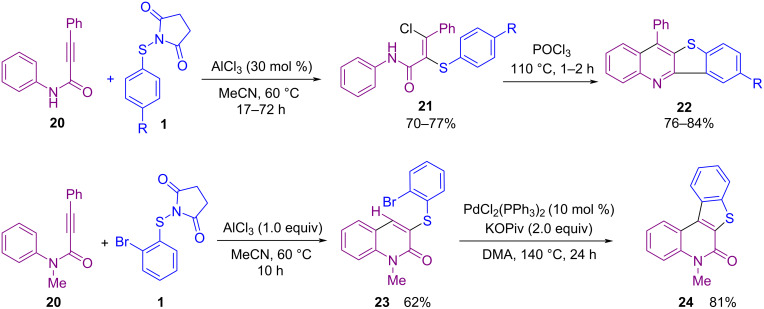
Synthetic utility of AlCl_3_‑catalyzed cyclization of *N*‑arylpropynamides with *N*‑sulfanylsuccinimides.

In addition to the use of *N*-(alkyl/arylthio)succinimides in the sulfenylation of organic compounds, *N*-(alkyl/arylthio)phthalimides are also considered good candidates for this purpose. In 2017, Sahoo and co-workers established a method for intramolecular annulation of *N*-(arylthio)phthalimides **14** and *N*-(arylthio)succinimides **1** with alkynes **15** in the presence of AlCl_3_ as an efficient Lewis acid catalyst (Scheme 10) [50]. In the procedure, oxidative cleavage of one S–N bond and 1,2-sulfur migration afforded π-conjugated 6-substituted 2,3-diarylbenzo[*b*]thiophenes **16**. A plausible mechanism is shown in Scheme 11. The coordination of AlCl_3_ with the phthalimide/succinimide unit of **1** or **14**, caused polarization of the S–N bond and produced an electrophilic intermediate **I**. Through the nucleophilic attack of the alkyne on **I**, cation **II** was generated, leaving Al-coordinated phthalimide/succinimide **III**. Finally, 4-*endo*-trig spirocyclization of **II** rendered the unstable intermediate **IV**, which underwent a ring expansion and 1,2-sulfur migration and subsequent deprotonation/aromatization to deliver product **16**. Another work in the use of AlCl_3_ for cyclization of *N*‑arylpropynamides **17** with *N*‑sulfanylsuccinimides **1** was described by Gao and Zhou et al. (Scheme 12) [51]. Annulation in the presence of AlCl_3_ led to 3‑sulfenylquinolin-2-ones **18**, while the addition of methanol into the reaction mixture gave 3-sulfenylazaspiro[4,5]trienones **19** as the target products. On the other hand, when free N–H alkynamides **20** were treated with *N*-sulfanylsuccinimides **1** in the presence of AlCl_3_, the coupling chlorinated product **21** was detected, which with POCl_3_ gave the cyclized product **22**. Also, the synthesis of benzo[*b*]thieno[2,3-*c*]quinolone **24** as an anticancer molecule was demonstrated in this approach (Scheme 13).

**Scheme 14:**
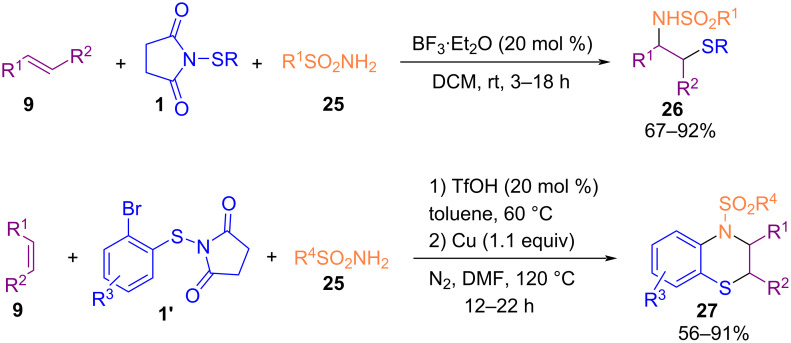
Sulfenoamination of alkenes with sulfonamides and *N*-sulfanylsuccinimides.

An intermolecular sulfenoamination of alkenes **9** with sulfonamides **25** as the nitrogen source and *N*-thiosuccinimides **1** as the sulfur source was reported by Gao and Liu et al. (Scheme 14) [52]. Highly regio- and diastereoselective β-sulfonylamino sulfides **26** were obtained from alkenes **9**, *N*-thiosuccinimides **1**, and sulfonamides **25** in the presence of 20 mol % BF_3_·Et_2_O. While the transformation in the presence of *N*-(2-bromophenylthio)succinimide **1’** and copper catalyst led to intermolecular sulfenoamination of alkenes and subsequent C–N coupling to produce dihydrobenzothiazine structures **27** in a one-pot manner. Furthermore, deprotection of the amine unit by K_2_CO_3_ and Na metal was performed in this work.

**Scheme 15:**
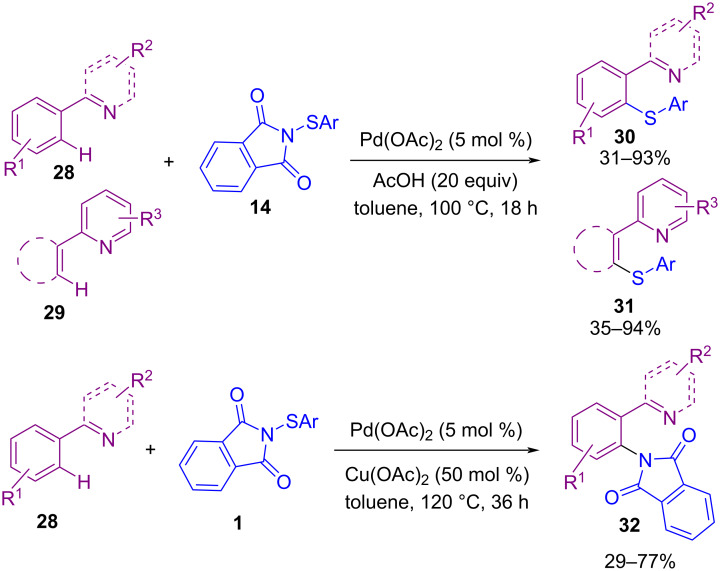
Lewis acid/Brønsted acid controlled Pd-catalyzed functionalization of aryl C(sp^2^)–H bonds.

**Scheme 16:**
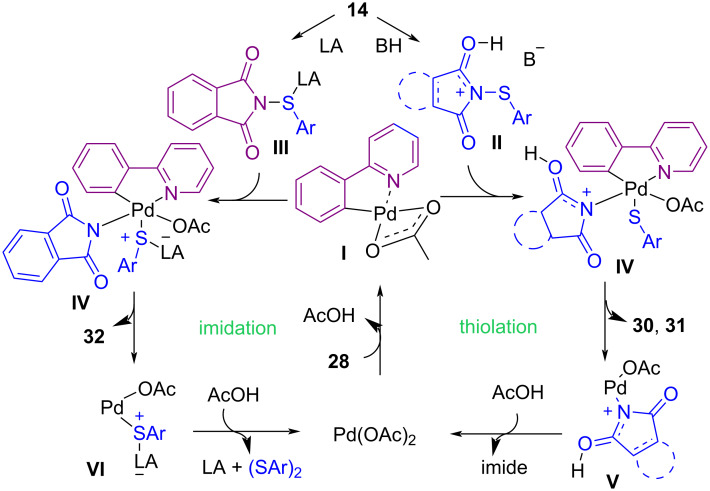
Possible mechanism for Lewis acid/Brønsted acid controlled Pd-catalyzed functionalization of aryl C(sp^2^)–H bonds.

In 2018, Anbarasan and Chaitanya developed an efficient approach for the C–H bond functionalization of aryl compounds containing a directing group using *N*-(thioaryl)phthalimides **14** in the presence of a palladium catalyst (Scheme 15) [53]. The thiolation occurred in the presence of Pd(OAc)_2_ and acetic acid (AcOH) as a Brønsted acid, whereas i(a)midation was achieved by using Pd(OAc)_2_ as catalyst and Cu(OAc)_2_ as a Lewis acid. A possible mechanism for this chemodivergent C–H activation is depicted in Scheme 16. First, Pd catalyzed the formation of palladacycle **I**. Oxidative addition of AcOH activated the N–S bond in **II**, which reacted with **I** to obtain **IV**, followed by C–S reductive elimination to give the thiolated product **30** or **31**. On the other hand, the interaction of **I** with Cu(OAc)_2_ activated the N–S bond in **III** to afford **IV**, which was subjected to C–N reductive elimination to deliver the imidated product **32**.

**Scheme 17:**
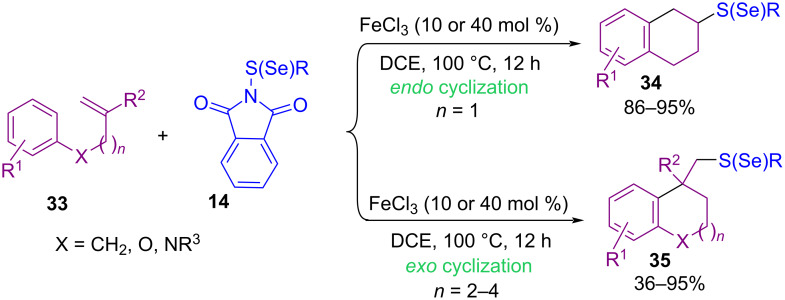
FeCl_3_-catalyzed carbosulfenylation of unactivated alkenes.

**Scheme 18:**
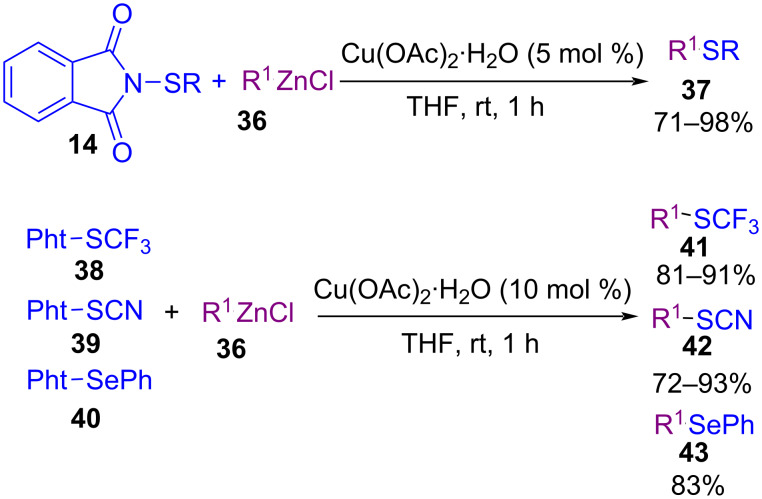
Copper-catalyzed electrophilic thiolation of organozinc halides.

**Scheme 19:**
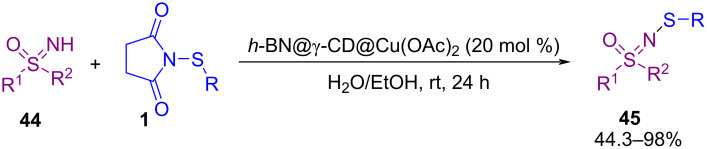
*h*-BN@Copper(II) nanomaterial catalyzed cross-coupling reaction of sulfoximines and *N*‑(arylthio)succinimide.

In 2018, an Fe-catalyzed carbosulfenylation and carboselenylation **33** of alkenes with *N*-(thio/seleno)phthalimides **14** was introduced by Lv and Li (Scheme 17) [54]. The use of Lewis acids, such as AlCl_3_, ZnCl_2_, InCl_3_, Fe(OTf)_2_ and Fe(acac)_3_ was not beneficial. However, BF_3_·OEt_2_, SnCl_4_, and TMSOTf resulted in good chemical yields. In the transformation, the selectivity of the *endo* or *exo* cyclization depended on the atom number of the chain between alkene and arene, leading to the formation of 6-, 7-, or 8-membered rings. In addition to *N*-(thio)phthalimides, benzenesulfenyl chloride as a sulfenylating source gave the target product in 93% yield. Knochel and co-workers found that copper acetate can catalyze the cross-coupling reaction between (hetero)aryl, alkyl and benzylic zinc halides **36** with *N*-thiophthalimides **14** (Scheme 18) [55]. Various metal catalysts, including CrCl_2_, CoCl_2_, NiCl_2_, MnCl_2_, FeCl_2_, Fe(acac)_3_ and copper salts such as Cu(OAc)_2_, CuBr_2_, CuBr, CuCl_2_, and CuCN·2LiCl were evaluated in this coupling reaction, in which Cu(OAc)_2_ showed highest product yields. Moreover, phthalimides with SCF_3_, SCN, and SePh groups also worked well in this approach. Because of the low reactivity of these phthalimides, 10 mol % of catalyst was required. Cross-coupling reaction of sulfoximines **44** with *N*‑(arylthio)succinimides **1** catalyzed by a nanomaterial containing hexagonal boron nitride (*h*-BN) and γ-cyclodextrin-supported copper(II) acetate (*h*-BN@γ-CD@Cu(OAc)_2_) was developed by Guo and Wu et al. (Scheme 19) [56]. Employment of a reusable heterogeneous nanomaterial, mild reaction conditions, avoiding the use of any additive, or base, and water/EtOH as a green solvent system were the advantages of this new method. *N*-Sulfenyl sulfoximines **45** were synthesized as coupling products in moderate to excellent yields.

**Scheme 20:**
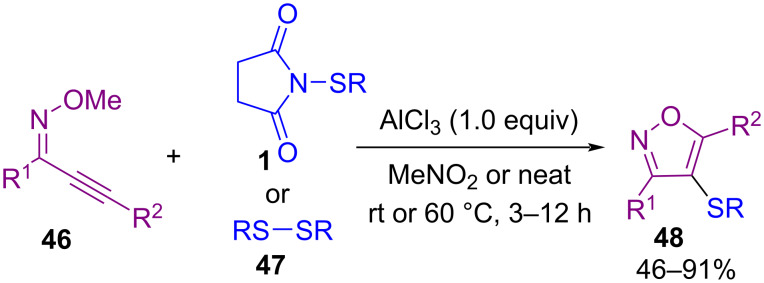
AlCl_3_‑mediated cyclization and sulfenylation of 2‑alkyn-1-one *O*‑methyloximes.

**Scheme 21:**
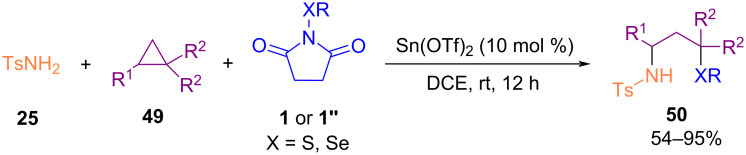
Lewis acid-promoted 2-substituted cyclopropane 1,1-dicarboxylates with sulfonamides and *N*-(arylthio)succinimides.

In 2019, Gao and Yang et al. disclosed a new protocol for the synthesis of 4‑aryl/alkylsulfenylisoxazoles **48** from sulfenylation of 2-alkyn-1-one *O*-methyloximes **46** with *N*-sulfenylsuccinimides **1** (Scheme 20) [57]. The transformation proceeded via an electrophilic cyclization and sulfenylation promoted by AlCl_3_. Dialkyl disulfides **47** were also well tolerated in this Lewis acid-mediated sulfenylation reaction in solvent-free conditions at room temperature. In the same year, a three-component reaction between highly substituted cyclopropanes **49**, sulfonamides **25** and *N*-(arylthio)succinimides **1** or *N*-(arylseleno)succinimides **1’’** was developed under a Lewis acid catalysis system. This reaction involves ring-opening of the substituted cyclopropane **49**, amination at the C1-site, and thiolation at the C3-site. In the transformation, sulfonamide acted as a nucleophile, chalcogensuccinimide as an electrophile, and cyclopropane as a zwitterion component (Scheme 21) [58].

**Scheme 22:**
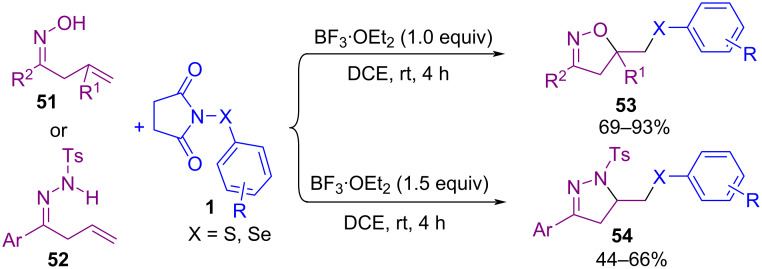
Lewis acid-mediated cyclization of β,γ-unsaturated oximes and hydrazones with *N*-(arylthio/seleno)succinimides.

**Scheme 23:**
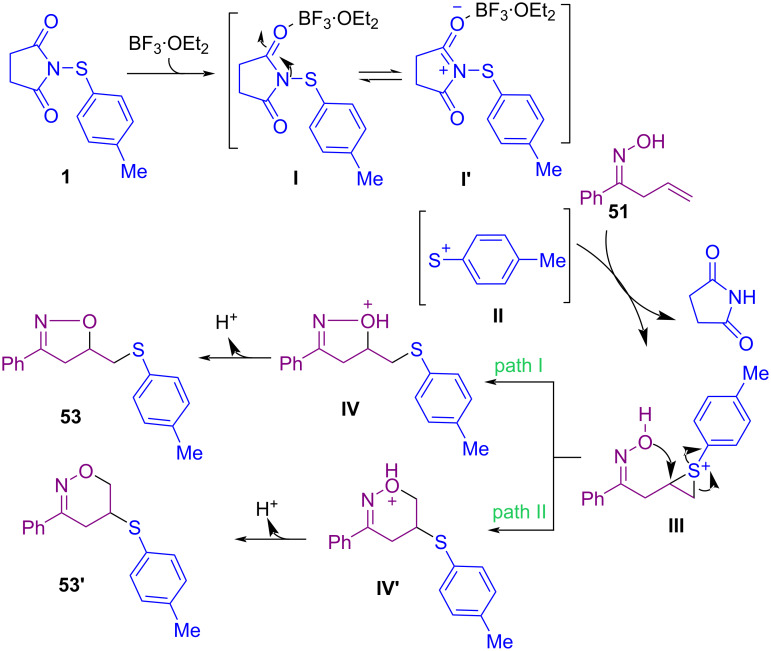
Credible pathway for Lewis acid-mediated cyclization of β,γ-unsaturated oximes with *N*-(arylthio)succinimide.

**Scheme 24:**
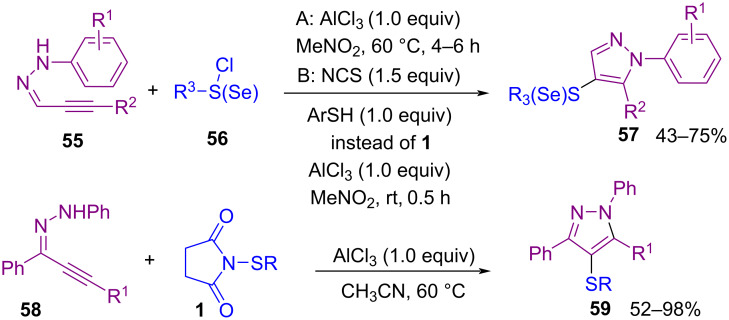
Synthesis of 4-chalcogenyl pyrazoles via chalcogenation/cyclization of α,β-alkynic hydrazones.

In 2020, a Lewis acid-mediated cyclization of β,γ-unsaturated oximes **51** and hydrazones **52** with *N*-(arylsulfenyl)succinimide **1** and *N*-(arylseleno)succinimide **1’’** was extended for the formation of isoxazoles **53** and dihydropyrazoles **54** as products (Scheme 22) [59]. A credible pathway for the production of isoxazole **53** is illustrated in Scheme 23. The interaction of **1** with BF_3_·Et_2_O resulted in intermediate **I** that is in balance with **I’**. Cleavage of the N–S bond of **I** afforded cationic species PhS^+^ **II**, which activated the double bond of **51** to give the three-membered ring **III**. Afterwards, intermediate **IV** was formed by an intramolecular ring opening of **III** (path I) and presumably produced **IV'** by path II, which through deprotonation delivered products **53** and **53'** respectively. In the meantime, another Lewis acid-promoted construction of 4-chalcogenylated pyrazoles **57** and **59** was carried out starting from α,β-alkynic hydrazones **55** (Scheme 24) [60]. In the procedure, α,β-alkynic hydrazones were subjected to S- or Se-electrophiles **56** and cyclization reaction. Additionally, NCS and ArSH produced the S-electrophile for the cyclization reaction with arylpropynal hydrazones. Also, the reaction of 1-(1,3-diphenylprop-2-yn-1-ylidene)-2-phenylhydrazine **58** as the substrate with *N*-sulfenylsuccinimides **1** afforded fully substituted pyrazoles **59** in up to 98% yield.

**Scheme 25:**
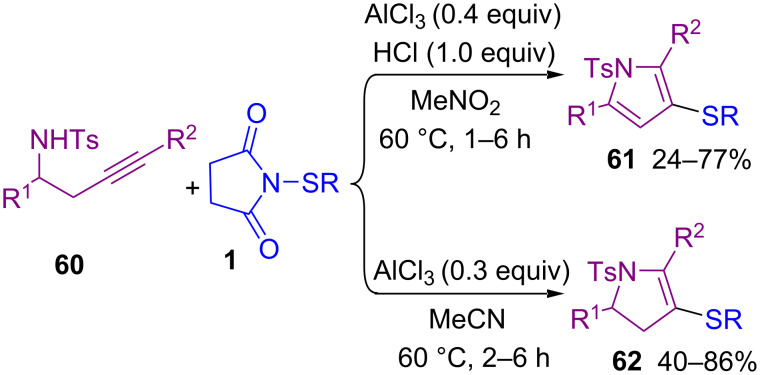
Controllable synthesis of 3-thiolated pyrroles and pyrrolines.

**Scheme 26:**
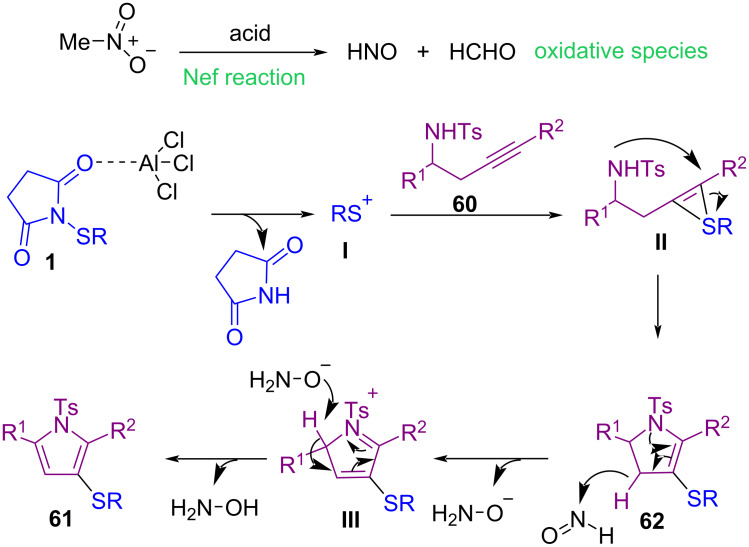
Possible mechanism for controllable synthesis of 3-thiolated pyrroles and pyrrolines.

In 2021, a solvent-controllable approach for the construction of 3-thiolated pyrroles **61** and pyrrolines **62** from propargylic tosylamides **60** and *N*-thiosuccinimides **1** was described by Gao′s group (Scheme 25) [61]. When AlCl_3_ as the Lewis acid catalyst and nitromethane as the solvent were used, a series of 3-thiolated pyrrole products **61** were detected, and 3-thiolated pyrrolines **62** were obtained by changing the reaction solvent to MeCN. Also, organic fluorophore compounds such as benzothienopyrrole and bis-thiolated boron dipyrromethene can be achieved from 3-thiolated pyrroles. Mechanistic studies showed that the oxidative species HNO and HCHO were generated through a Nef reaction in MeNO_2_ under acidic conditions. In the meantime, **1** was activated by AlCl_3_ to form sulfenium cation **I**, which induced an intramolecular cyclization of **60** to produce pyrroline **62**. In MeCN solvent, 3-thiolated pyrroline **62** was stable and could be isolated, but in MeNO_2_, in the presence of the HNO species, the pyrroline structure could oxidize and aromatize to the pyrrole ring **61** (Scheme 26).

**Scheme 27:**
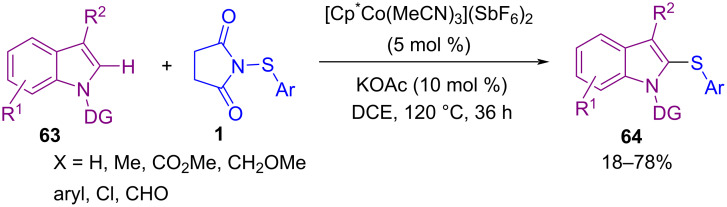
Co-catalyzed C2-sulfenylation and C2,C3-disulfenylation of indole derivatives.

**Scheme 28:**
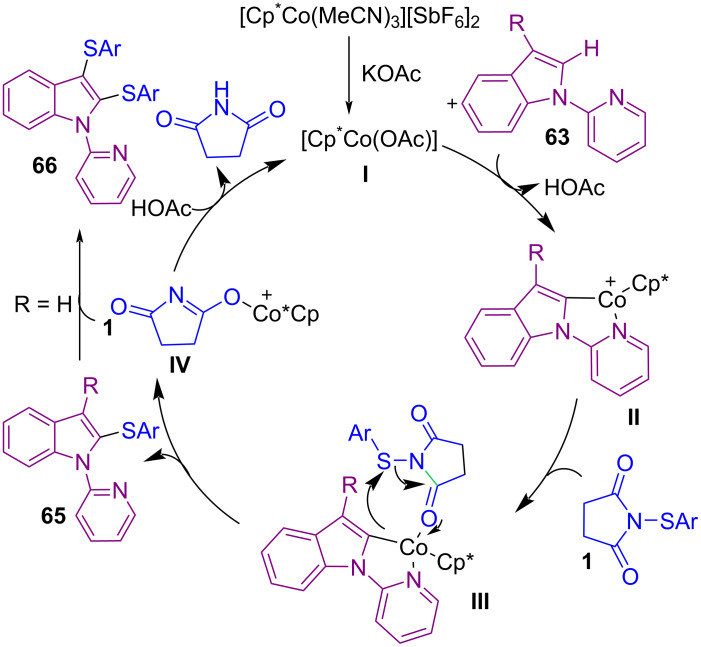
Plausible catalytic cycle for Co-catalyzed C2-sulfenylation and C2,C3-disulfenylation of indoles.

**Scheme 29:**
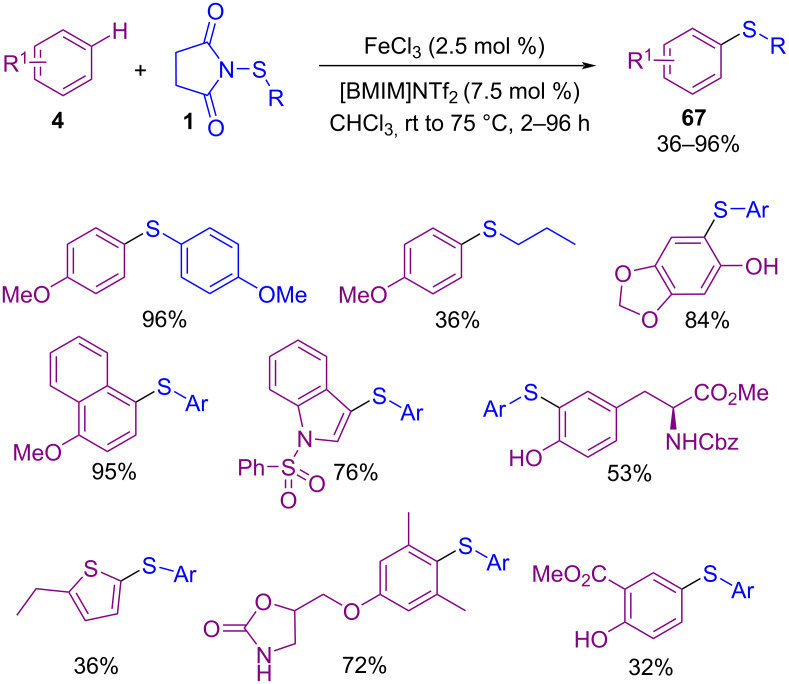
C–H thioarylation of electron-rich arenes by iron(III) triflimide catalysis.

In 2021, Anbarasan and co-workers were able to obtain a diverse range of sulfenylated products **64** in a Co-catalyzed C2-sulfenylation and C2,C3-disulfenylation of indole derivatives with *N*-(arylsulfenyl)succinimide **1** (Scheme 27) [62]. The reaction involves the formation of active cobalt species **I** from the interaction of KOAc with the cobalt pre-catalyst. Treatment of **I** with **63** resulted in the five-membered cobaltocycle complex **II**. Next, coordination of **1** to **II** gave **III**, followed by intramolecular nucleophilic trapping of the electrophilic SAr unit to furnish C2-sulfenylated product **65** and Co-complex **IV**. At last, active cobalt species **I** regenerated from **IV** in the presence of AcOH. It should be noted that when R = H, C2-sulfenylated product **65** may be sulfenylated via a thermal electrophilic aromatic substitution to provide C2,C3-disulfenylated product **66** (Scheme 28). In the same year, Sutherland and Dodds disclosed a protocol for the C–H bond thioarylation of electron-rich arenes **4** like anisoles, acetanilides, phenols, and *N*-heterocycles in the presence of Fe(III) Lewis acid and ionic liquid [BMIM]NTf_2_ as an effective catalysis system (Scheme 29) [63]. Kinetic studies in this cross coupling-reaction indicated that *N*-(arylthio)succinimides **1** with electron-deficient arene **4** undergoe thioarylation catalyzed by Fe(NTf_2_)_3_. Related molecules bearing an electron-rich arene showed an autocatalytic pathway that is enhanced due to the Lewis basic character of the final product.

**Scheme 30:**
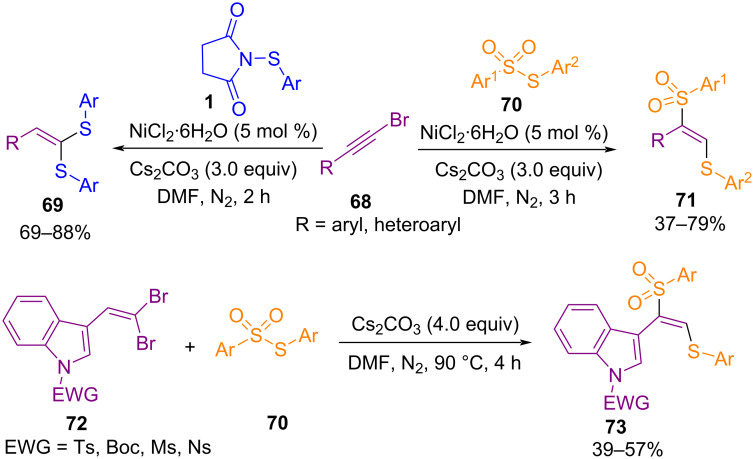
Difunctionalization of alkynyl bromides with thiosulfonates and *N*-arylthio succinimides.·

**Scheme 31:**
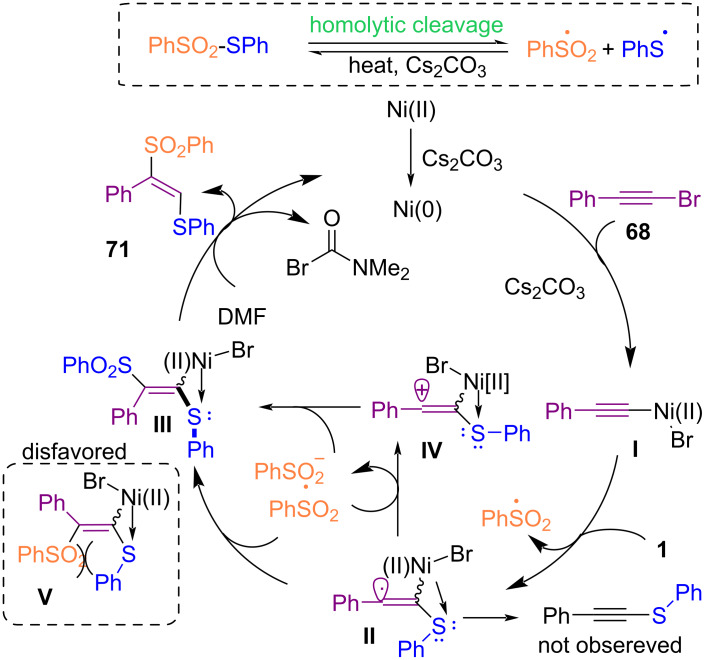
Suggested mechanism for difunctionalization of alkynyl bromides with thiosulfonates and *N*-arylthio succinimides.

Reddy and co-workers developed a simple method for the preparation of 1,2-thiosulfonylethenes **71** and 1,1-dithioethenes **69** in the presence of a nickel catalyst (Scheme 30) [64]. Various alkynyl bromides **68** as starting materials reacted with thiosulfonates **70** and *N*-arylthiosuccinimides **1** as thiolating reagents. 1,2-Thiosulfonylethenes **71** were obtained via vicinal thiosulfonylation. However, in the case of 1,1-dithioethenes **69**, germinal disulfenylation occurred. In addition, 1,2-difunctionalization of indole-derived 1,1-bromoalkenes **72** was also investigated in the presence of Cs_2_CO_3_ without the need of a metal catalyst. The synthetic applicability of the procedure was demonstrated by a gram-scale synthesis of the 1,2-thiosulfonylethene product. A possible mechanism for the formation of 1,2-thiosulfonylethenes is shown in Scheme 31. Initially, homolytic cleavage of thiosulfonate **70** generated PhS· and PhSO_2_· radicals. The reduction of Ni(II) to Ni(0) in the presence of Cs_2_CO_3_ and the reaction with **68** formed alkynyl-Ni species **I**. Then, the PhS· radical reacted with **I** to generate alkenyl radical **II**, which can react with the PhSO_2_· radical to obtain intermediate **III**. Radical **II** underwent oxidation with PhSO_2_· to form alkenyl cation **IV** and PhSO_2_^−^. At last, H-abstraction from DMF delivered product **71** and the Ni(0) species to continue the catalytic cycle.

**Scheme 32:**
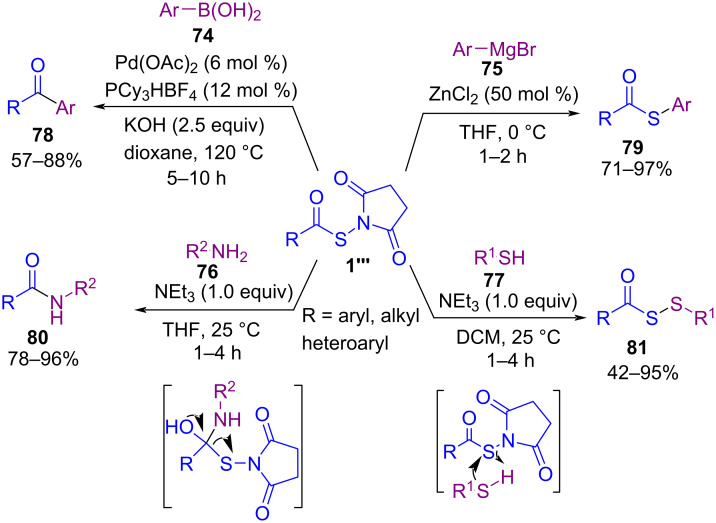
Synthesis of thioesters, acyl disulfides, ketones, and amides by *N*-thiohydroxy succinimide esters.

**Scheme 33:**
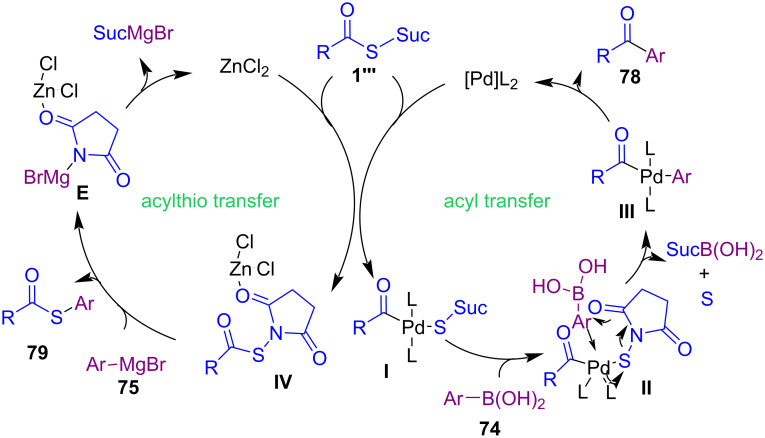
Proposed mechanism for metal-catalyzed selective acylation and acylthiolation.

In 2022, Gao and co-workers introduced a new protocol for the preparation of thioesters, acyl disulfides, ketones, and amides starting from *N*-thiohydroxy succinimide esters (NTSEs) **1’’’**, which can serve as the acylthiolating and acylating source (Scheme 32) [65]. First, they synthesized a series of *N*-thiohydroxy succinimide esters by treating potassium thiolates with *N*-chlorosuccinimide in MeCN at room temperature for 20 min. *N*-Thiohydroxysuccinimide esters were obtained in up to high yields (21–83%). In the next phase, they performed the reaction of NTSEs with different nucleophiles, according to hard acyl and soft acylthio electrophilic sites contained in the NTSEs to selectively transfer the acyl or acylthio moieties. Arylboronic acids **74** and amines **76** were suitable for the acyl transfer and led to ketones **78** and amides **80** as the desired products. While, Grignard reagents **75** and thiols **77** acted as soft nucleophiles and resulted in thioesters **79** and acyl disulfides **81**, respectively. It should be noted that the use of a palladium catalyst was essential for the cross-coupling reaction between **1’’’** and **74**. Also, the presence of ZnCl_2_ could facilitate the cleavage of the N–S bond. In the case of amines and thiols, there was no need for a metal catalyst for the formation of S–N and S–S bonds. A plausible mechanism for the metal-catalyzed acylation and acylthiolation is illustrated in Scheme 33. Firstly, oxidative addition of palladium to the C–S bond of NTSE **1’’’** afforded intermediate **I**. The transmetalation from boron to palladium led to intermediate **III**, followed by reductive elimination to yield ketone **78**. In the acylthiolation cycle, the azaphilic ZnCl_2_ activated NTSE **1’’’** via N–Zn coordination to facilitate the leaving ability of succinimide. Then, nucleophilic substitution of arylmagnesium bromide **75** to intermediate **IV** provided thioester **79**.

**Scheme 34:**
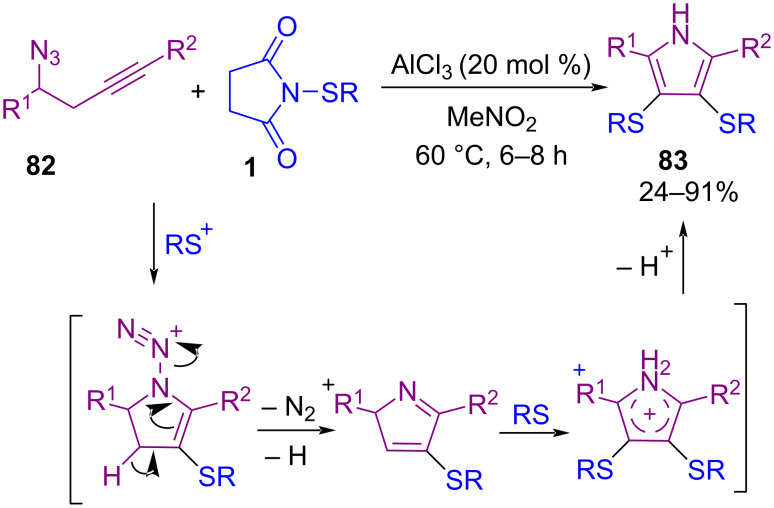
AlCl_3_-catalyzed synthesis of 3,4-bisthiolated pyrroles.

In 2022, Gao and co-workers demonstrated bisulfenylation/cyclization of homopropargylic azides **82** with *N*-thiosuccinimides **1** in the presence of AlCl_3_ as the catalyst, 3,4-bisthiolated pyrroles **83** were obtained as the desired products in moderate to high yields (Scheme 34) [66]. The reaction involves the Lewis acid-catalyzed first thiolation and intramolecular cyclization of propargyl azides the removal of N_2_ and a proton. Subsequently, monothiolated perroles were subjected to the second thiolation process to prepare 3,4-bisthiolated pyrroles. Cyclic voltammetry and DFT calculations revealed that the 3,4-bisthiolated pyrroles **83** contained higher HOMO orbital energies, and lower band gaps compared to the unsubstituted parent 2,5-diphenylpyrrole.

#### Organocatalyzed sulfenylation by *N*-(sulfenyl)succinimides/phthalimides

**Scheme 35:**
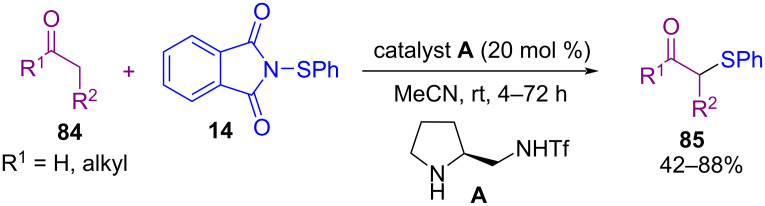
α-Sulfenylation of aldehydes and ketones.

In 2004, direct sulfenylation of a series of aldehydes and ketones **84** with *N*-(phenylthio)phthalimide (**14**) by using an organocatalyst was reported by Wang and co-workers (Scheme 35) [67]. Several orgnocatalysts, such as piperidine, and pyrrolidine derivatives were evaluated for the coupling reaction, in which pyrrolidine trifluoromethanesulfonamide **A** was selected as the best catalyst for this purpose. It is noteworthy that the use of diphenyl disulfide as a sulfenylating agent was not effective in this protocol. *N*-(Aryl/alkylthio)phthalimide as an efficient sulfenylating reagent could also react with indoles to produce 3-thioindoles in the presence of 0.5 mol % of MgBr_2_, as a Lewis acid [68]. Moreover, sulfenylation of ketoximes and secondary nitro compounds toward *N*-arenesulfenyl ketimines occurred by applying *N*-(phenylthio)phthalimide [69].

**Scheme 36:**
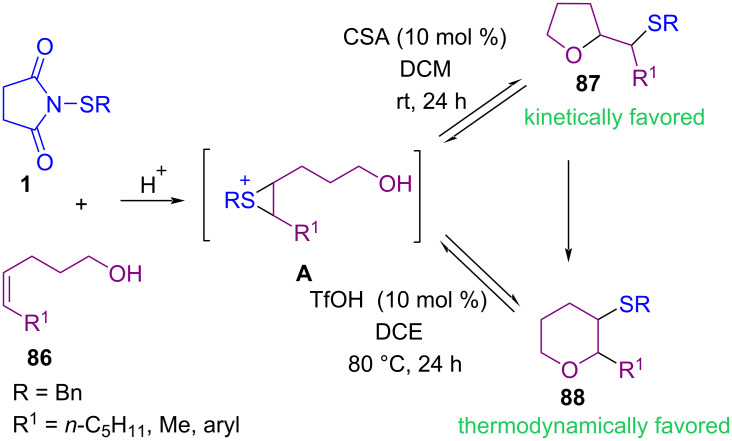
Acid-catalyzed sulfetherification of unsaturated alcohols.

**Scheme 37:**
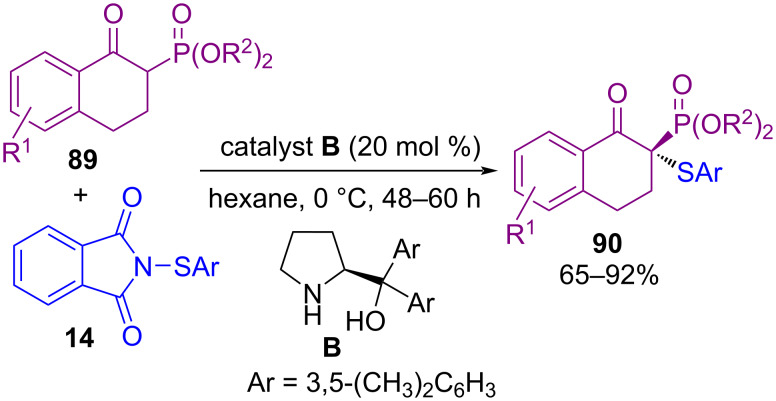
Enantioselective sulfenylation of β-keto phosphonates.

In 2011, Shi et al. developed a method for the sulfenylation of unsaturated alcohols **86** by *N*-(benzylthio)succinimide **1** access to tetrahydrofurans **87** and tetrahydropyrans **88** (Scheme 36) [70]. In this protocol, by controlling acid catalyst (camphorsulfonic acid (CSA) or trifluoromethanesulfonic acid (TfOH)), two different products were achieved and tetrahydrofurans **87** could be converted to tetrahydropyrans **88** by stereoselective rearrangement. In the same year, Zhu and Cheng et al. developed a convenient approach for the thiolation of β-keto phosphonates **89** by using *N*-(arylthio)phthalimides **14** under α,α-diaryl-ʟ-prolinols **B** organocatalytic system (Scheme 37) [71].

**Scheme 38:**
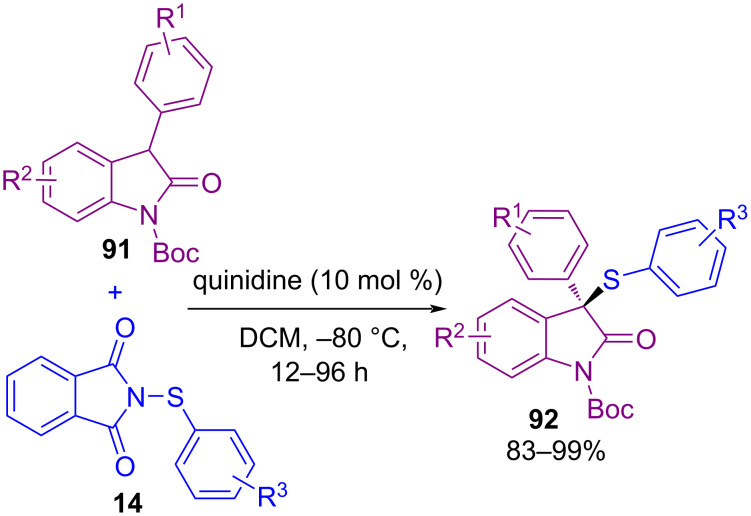
Organocatalyzed sulfenylation of 3‑substituted oxindoles.

Sulfenylation of 3-aryloxindoles **91** with *N*-(arylsulfenyl)phthalimides **14** as the electrophilic sulfur reagents resulted in thiolated products **92** up to 99% ee, in the presence of quinidine as the organocatalyst (Scheme 38) [72]. For the study of enantioselectivity of products, different *N*-substituted oxindoles with H, Me, phenyl, and benzyl groups were investigated. As the size of *N*-protecting groups increased, the percentage of enantioselectivity decreased, where in the case of NH-oxindoles, the product was achieved with only 6% ee. Another sulfenylation at the 3-position of unprotected oxindoles with *N*-(phenylthio)phthalimide was reported by Feng et al. [73]. A chiral *N*,*N′*-dioxide-Sc(OTf)_3_ complex as a catalyst and a Brønsted base were applied in the procedure. It is interesting to note that in such a method, sulfenylation of NH-oxindoles resulted in the thiolated products with excellent enantioselectivities (up to 99% ee).

**Scheme 39:**
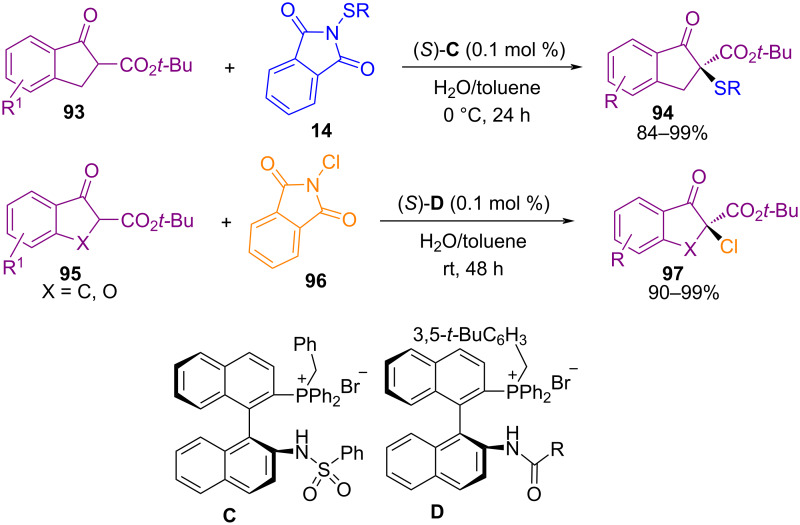
Sulfenylation and chlorination of β-ketoesters.

In 2013, sulfenylation and chlorination of β-ketoesters **93**, and **95** with *N*-(arylthio)phthalimide **14** and *N*-chlorophthalimide (**96**) under phase-transfer conditions was developed by Maruoka and co-workers (Scheme 39) [74]. The presence of chiral bifunctional catalysts **C** and **D** with the amide, or sulfonamide moieties could improve the enantioselectivity. Also, the heterogeneous medium coming from H_2_O and toluene was beneficial for the progress of the transformation.

**Scheme 40:**
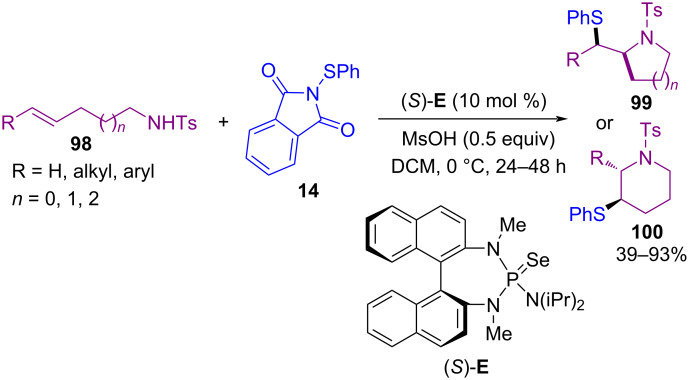
Intramolecular sulfenoamination of olefins.

**Scheme 41:**
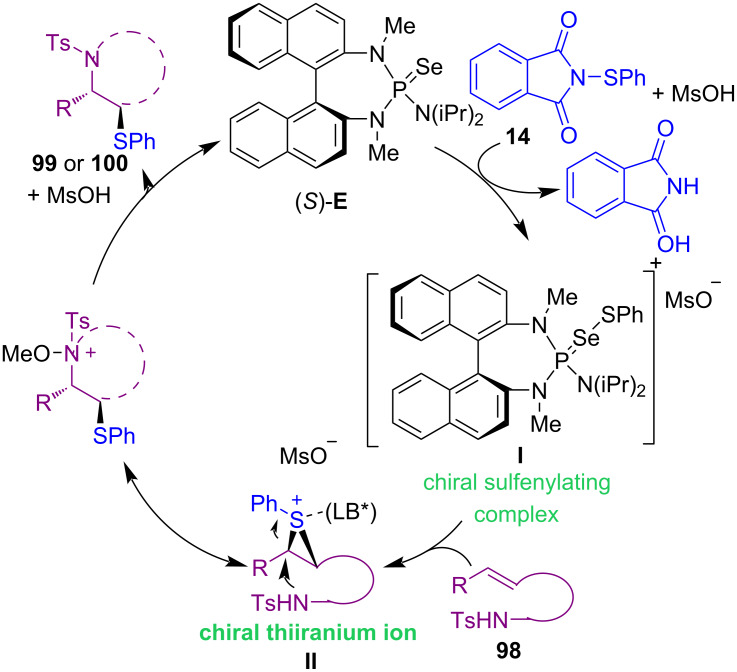
Plausible mechanism for intramolecular sulfenoamination of olefins.

**Scheme 42:**
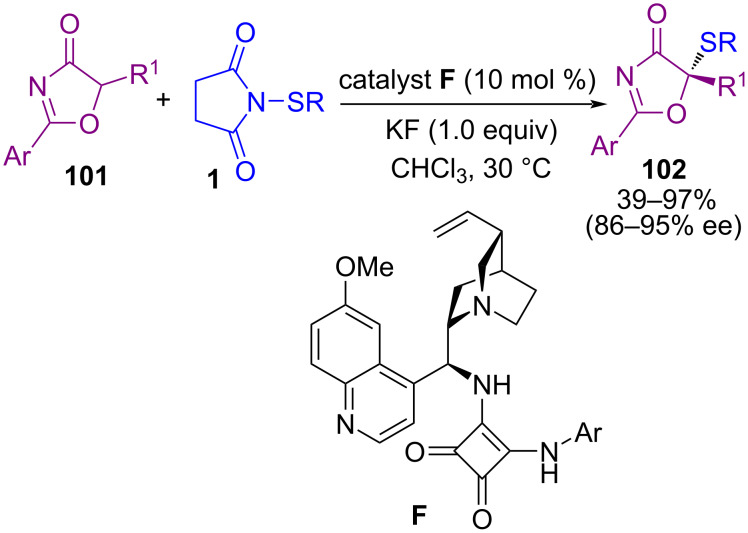
α-Sulfenylation of 5*H*-oxazol-4-ones.

In 2014, Denmark and Chi successfully synthesized a wide variety of pyrrolidines **99**, piperidines **100**, and azepanes via intramolecular sulfenoamination of olefins **98** (Scheme 40) [75]. The reduction of *endo* to *exo* ratio was either related to the electron density of the alkene or the steric effect of a substituent. The tether lengths could affect the cyclization. For example, the two-carbon-tethered substrate completely showed *endo* selectivity, while the four-carbon-tethered substrate exclusively led to azepane. A possible mechanism was suggested for this Lewis base catalysis system. Methanesulfonic acid (MsOH) activated reagent **14**, which coordinated with the Lewis base (*S*)-**E**, to form complex **I**. Then, the transfer of the sulfenium ion to the alkene resulted in chiral thiiranium ion **II**. Capture of the thiiranium ion by the tosylamide and deprotonation led to the final product **99** or **100** (Scheme 41). Through the coupling reaction of *N*-(aryl/alkylthio)succinimides **1** with 5*H*-oxazol-4-ones **101** in the presence of an organocatalyst named cinchona alkaloid-derived squaramide **F**, a series of α-sulfenylated products **102** were obtained in moderate to excellent yields with good to excellent enantioselectivities (Scheme 42) [76]. It should be noted that the authors did not define the exact role of the organocatalyst in the reaction mechanism.

**Scheme 43:**
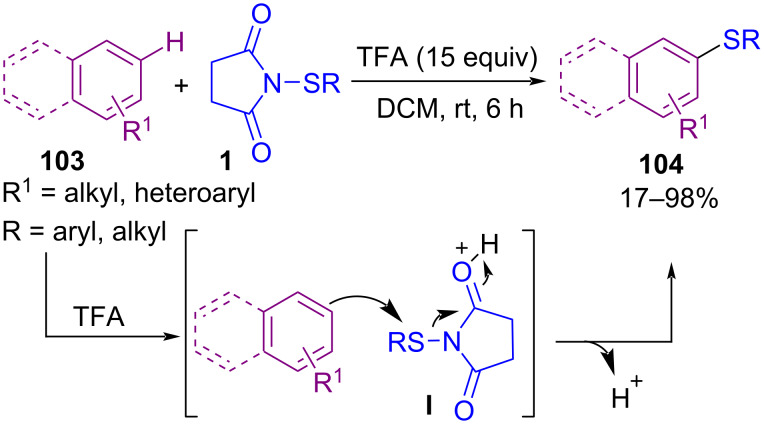
Metal-free C–H sulfenylation of electron-rich arenes.

**Scheme 44:**
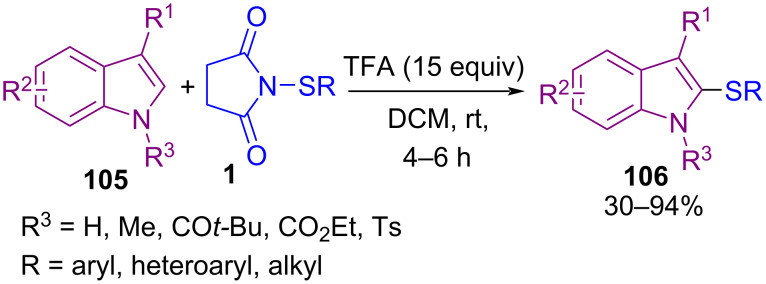
TFA-promoted C–H sulfenylation indoles.

**Scheme 45:**
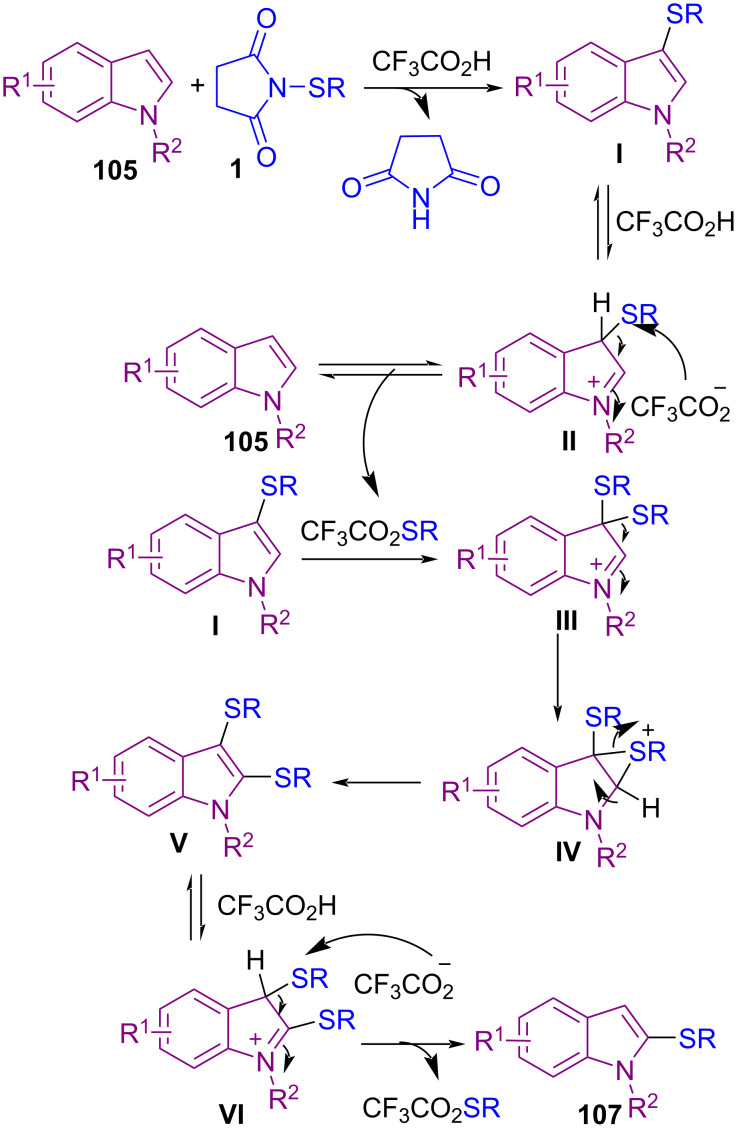
Proposed mechanism for TFA-promoted C–H sulfenylation indoles.

Transition-metal-free C–H sulfenylation of electron-rich arenes **103** by *N*-(alkyl/arylthio)succinimides **1** led to aryl sulfides **104** (Scheme 43) [77]. The cross-coupling reaction involves protonation of the succinimide moiety by trifluoroacetic acid (TFA) to create electrophilic thio intermediate **I**. Nucleophilic attack of arene **103** on **I** led to target product **104**. Also, TFA-catalyzed C–H sulfenylation at the C2-position of protected and unprotected indoles **105** to form 2-thioindoles **106** (Scheme 44) [78]. The reaction initiated with TFA-promoted electrophilic addition of **1** to **105** towards C3-sulfenylated indole **I**, which was protonated by TFA, led to intermediate **II**. Then, CF_3_CO_2_SR, which was produced in the previous step, as a sulfenylating reagent, reacted with **I** to form the 3,3-bis-sulfide indolenium **III**. The migration of a sulfide group to the C2-site of indole, generated 2,3-disubstituted indole **V**. Protonation of **V** resulted in indolenium intermediate **VI**. Finally, desulfenylation of **VI** by anion CF_3_CO_2_, afforded 2-thioindole **107** (Scheme 45).

**Scheme 46:**
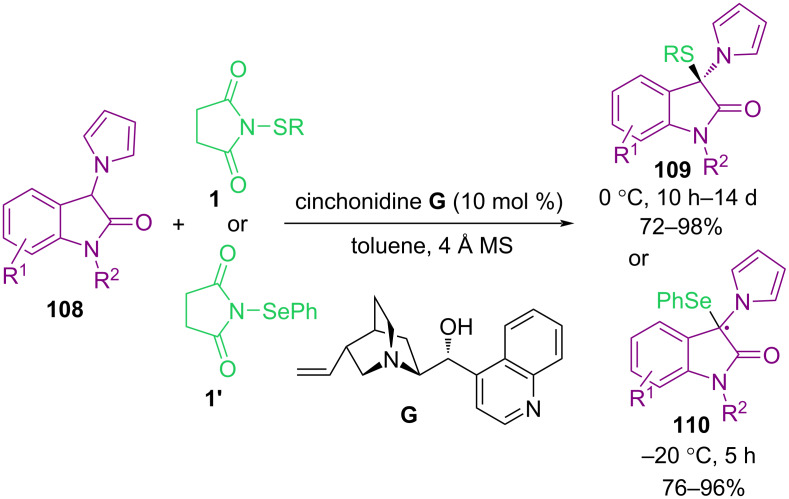
Organocatalyzed sulfenylation and selenenylation of 3-pyrrolyloxindoles.

**Scheme 47:**
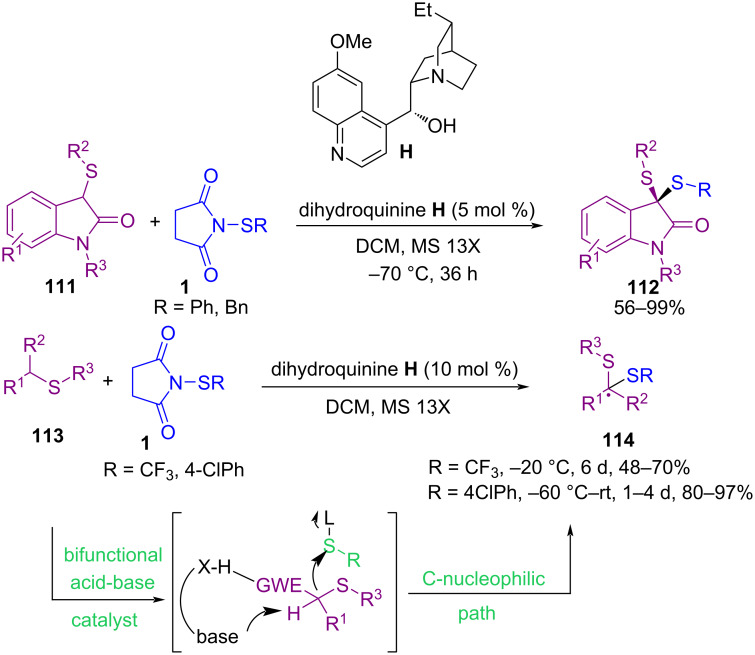
Organocatalyzed sulfenylation of *S*-based nucleophiles.

The enantioselective synthesis of a broad spectrum of 3-thio-3-pyrrolyloxindoles **109** and 3-seleno-3-pyrrolyloxindoles **110** via sulfenylation and selenenylation of 3-pyrrolyloxindoles **108** was described by Yuan′s research group in 2015 (Scheme 46) [79]. By testing several alkaloids as organocatalysts for the transformation, cinchonidine **G** proved to be the best catalyst for C–H sulfenylation and selenenylation of substrates in toluene at −20 or 0 °C. The reaction occurred in shorter times in the presence of *N*-(arylsulfanyl)succinimide, while the coupling reaction using *N*-(alkylsulfanyl)succinimide and *N*-(heteroarylsulfanyl)succinimide longed several days. The gram-scale synthesis demonstrated the practicality of this method. In the same year, sulfenylation of different types of *S*-based nucleophiles **111** and **113** with *N*-(organosulfanyl)succinimide **1** catalyzed by dihydroquinine as an easily available organocatalyst was reported by Zhou et al. (Scheme 47) [80]. This is the first example of the preparation of chiral dithioketals. The presence of the OH group was essential in dihydroquinine **H**. By changing OH into a OMe group, the enantioselectivity and the product yield were reduced. Although, the authors did not further explain the catalytic pathway.

**Scheme 48:**
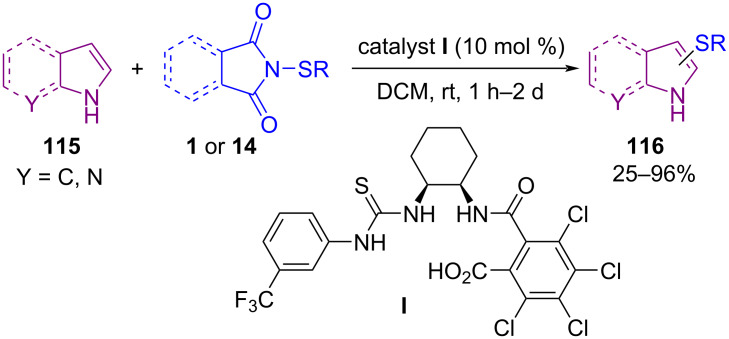
Conjugate Lewis base Brønsted acid-catalyzed sulfenylation of *N*-heterocycles.

**Scheme 49:**
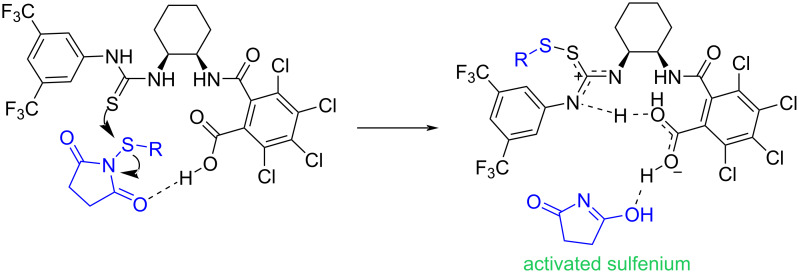
Mechanism for activation of *N*-sulfanylsuccinimide by conjugate Lewis base Brønsted acid catalyst.

**Scheme 50:**
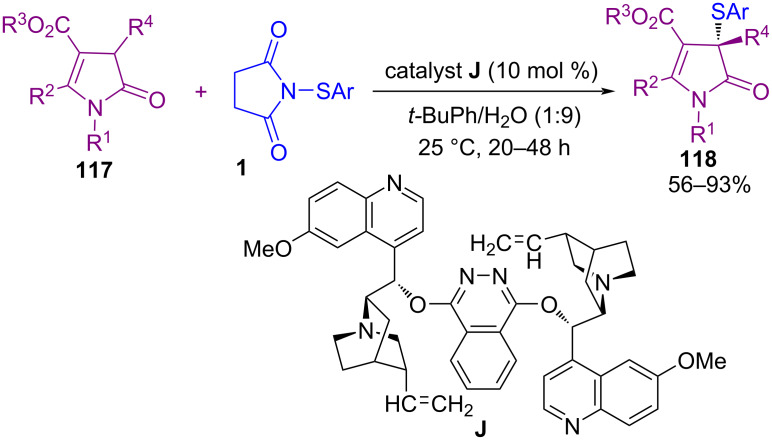
Sulfenylation of deconjugated butyrolactams.

**Scheme 51:**
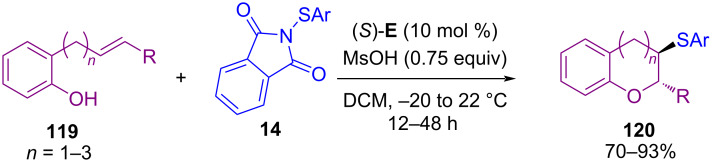
Intramolecular sulfenofunctionalization of alkenes with phenols.

The use of organocatalysts in sulfenylation of *N*-heterocyclic compounds was investigated by Gustafson′s group in 2017 (Scheme 48) [81]. In their work, a series of conjugate Lewis base Brønsted acid organocatalysts were evaluated for sulfenylation on C3, or C2 position of *N*-heterocycles **115**, including indoles, peptides, pyrrole, and 1-methyl-1*H*-pyrrolo[2,3-*b*]pyridine. The authors hypothesized a mechanism for the activation of *N*-sulfanylsuccinimides **1** or **14** by conjugate Lewis base Brønsted acid catalyst **I**, leading to the formation of an electrophilic sulfenium source (Scheme 49). The use of dimeric cinchona alkaloid **J** as another organocatalyst for α-sulfenylation of deconjugated butyrolactam substrates **117** with *N*-(arylsulfanyl)succinimides **1** demonstrated in Mukherjee′s work (Scheme 50) [82]. In the method, functionalized γ-lactams **102** were produced in aqueous media with high enantioselectivities. However, *N*-(alkylsulfanyl)succinimides and α-isobutyl containing butyrolactam did not work in this reaction. Another work by Denmark on intramolecular sulfenylation of alkenes **119** with phenols by using *N*-(arylthio)phthalimide **14** as a sulfur source was reported in the same year (Scheme 51) [83]. Benzopyrans **120** and benzoxepins were obtained in the presence of a Lewis base catalyst in good to high yields.

**Scheme 52:**
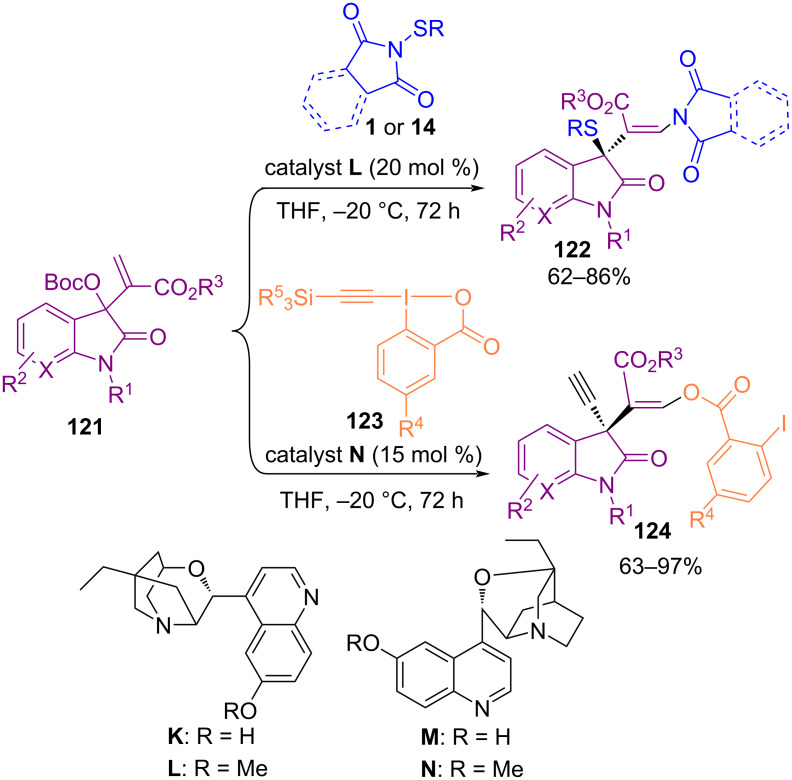
Organocatalytic 1,3-difunctionalizations of Morita–Baylis–Hillman carbonates.

**Scheme 53:**
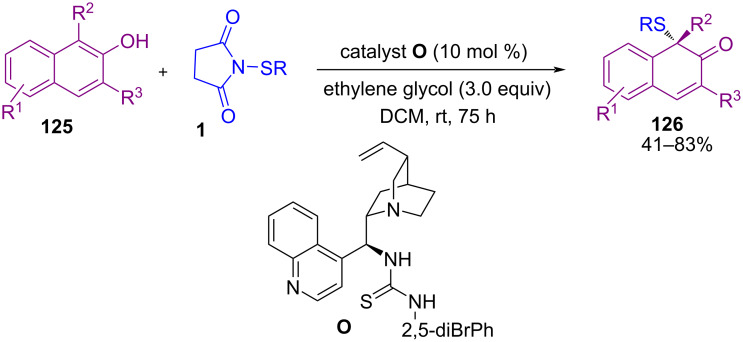
Organocatalytic sulfenylation of β‑naphthols.

In 2018, Liang and Chen et al. extended 1,3-difunctionalizations of a series of Morita–Baylis–Hillman carbonates from isatins by using a Lewis base catalytic system (Scheme 52) [84]. Screening several organocatalysts showed that the 1,3-oxo-ethynylation of starting materials with silylethynyl-1,2-benziodoxol-3(*1H*)-ones **123** was obtained by using catalyst **N**, while 1,3-aminosulfenylation with *N*-(aryl/alkylthio)imides **1** or **14** occurred in the presence of catalyst **L**. Meanwhile, Zhou and Chen′s research team was able to synthesize a broad range of enantioenriched naphthalenone structures **126** by utilizing another organocatalyst (Scheme 53) [85]. In the procedure, β‑naphthols **125** reacted with *N*-(arylthio)succinimide **1** or *N*-(arylthio)phthalimide **14** as the sulfenylating reagents in the presence of cinchona-derived thiourea **O** as a catalyst to afford the corresponding chiral naphthalenone products **126** under mild reaction conditions.

**Scheme 54:**
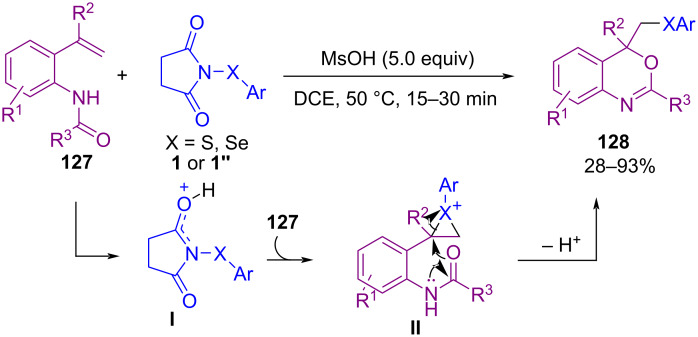
Acid-promoted oxychalcogenation of *o*‑vinylanilides with *N*‑(arylthio/arylseleno)succinimides.

**Scheme 55:**
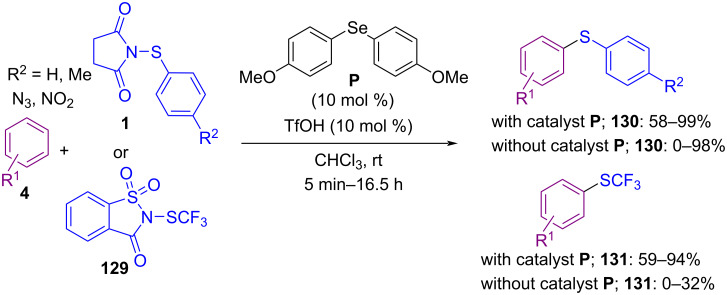
Lewis base/Brønsted acid dual-catalytic C–H sulfenylation of aryls.

Another work from Anbarasan and Chaitanya on the use of *N*-(arylsulfanyl)succinimide **1** and *N*-(arylseleno)succinimide **1’’** in an oxychalcogenation process was reported in 2018 (Scheme 54) [86]. In this method, they succeeded in applying methanesulfonic acid (MsOH) as a promoter for oxythiolation and oxyselenation of *o*-vinylanilides **127** through the formation of three-membered cyclic sulfonium ion **II** followed by ring-opening of sulfonium ion and intramolecular cyclization. The use of a Lewis base/Brønsted acid catalysis system for the sulfenylation of aromatic substrates **4** was reported by Gustafson et al. (Scheme 55) [87]. In the method, catalyst **P** acted as a Lewis base, where TfOH acted as a Brønsted acid. It is worth noting that coupling reactions without Lewis base catalyst **P** occurred in much lower yields. The mechanistic investigations showed that electron-rich sulfenyl groups can participate in an autocatalytic mechanism due to their Lewis basic nature. However, the electron-poor ones exhibited less autocatalysis effect requiring the use of the Lewis base catalyst **P**.

**Scheme 56:**
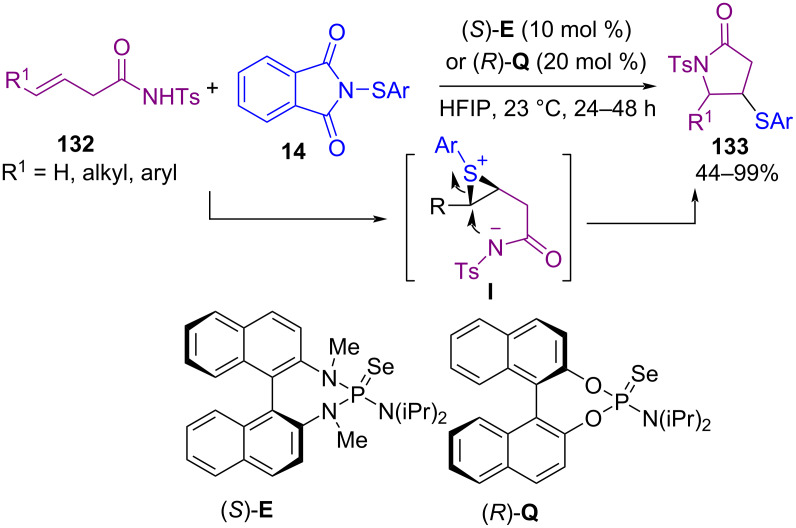
Lewis base-catalyzed sulfenoamidation of alkenes.

**Scheme 57:**
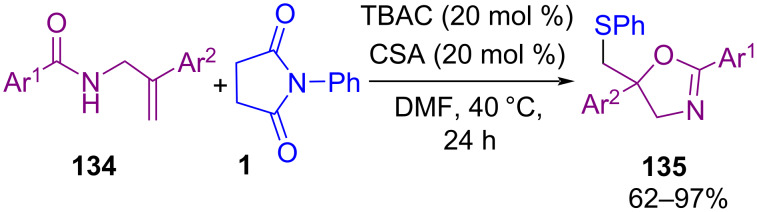
Cyclization of allylic amide using a Brønsted acid and tetrabutylammonium chloride.

In 2019, Denmark and Panger disclosed a novel method for the preparation of γ‑lactams **133** through the reaction of alkenes **132** with *N*-thiophthalimides **14** in the presence of Lewis base organocatalysts (Scheme 56) [88]. In this procedure, the cyclized products were obtained via the activation of the sulfur electrophile by a Lewis base to generate the thiiranium ion intermediate from the β,γ-unsaturated sulfonyl carboxamide. The attack of the sulfonamide nitrogen atom on this intermediate led to intramolecular cyclization. In 2020, electrophilic cyclization of allylic amides **134** using *N*-(phenylthio)succinimide **1** in the presence of camphorsulfonic acid (CSA) as a Brønsted acid and tetrabutylammonium chloride (TBAC) led to 5-[(phenylthio)methyl]oxazoline scaffolds **135** (Scheme 57) [89]. Combination of CSA/TBAC formed an efficient activator system for this sulfenylation/intramolecular cyclization.

**Scheme 58:**
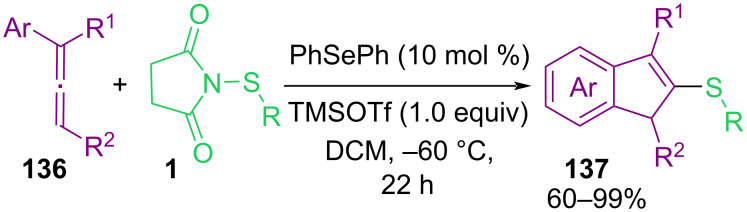
Catalytic electrophilic thiocarbocyclization of allenes with *N*-thiosuccinimides.

**Scheme 59:**
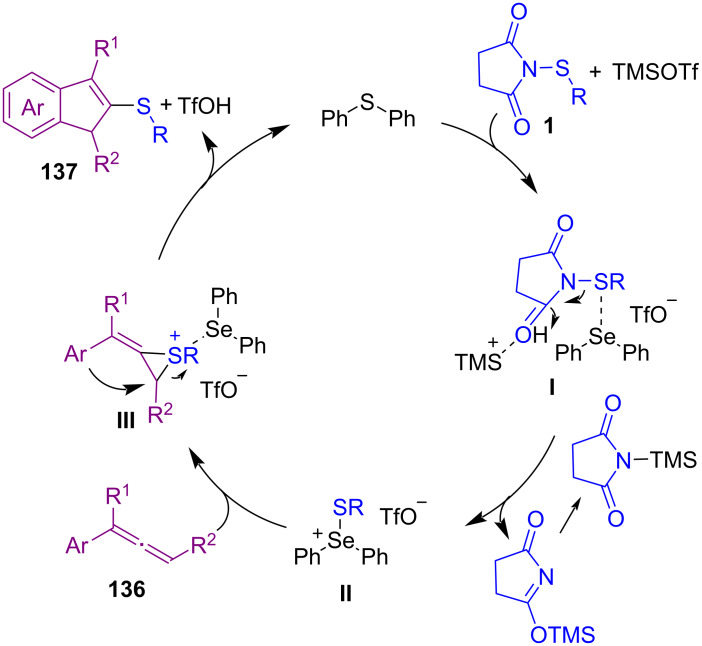
Suggested mechanism for electrophilic thiocarbocyclization of allenes with *N*-thiosuccinimides.

In the same year, Zhao and co-workers reported the thiocarbocyclization of allenes **136** with *N*-(organothio)succinimides **1** as electrophilic aryl/alkylthio reagents for the assembly of indene-based sulfide molecules **137** (Scheme 58) [90]. The Lewis basicity nature of PhSePh as a catalyst and the presence of Lewis acid TMSOTf improved the chemical yields. It is interesting to note that the reaction carried out at a lower temperature because of the high reactivity of allene **136**. When the reaction was performed at room temperature, no desired product was observed, and performing the reaction at 0 °C enhanced the regioselectivity but still in low yield. By further lowering the temperature to −60 °C the yield was increased. The authors suggested a possible mechanism for this organoselenium-catalyzed cyclization transformation involving activation of the electrophilic sulfur reagent by PhSePh with the assistance of TMSOTf to form transition state **I**. Intermediate **II** formed through capturing of sulfonium by selenium. Then, **II** reacted with **136** to give regioselective cyclic thiiranium ion **III**. Nucleophilic attack of the aromatic ring on the thiiranium ion moiety furnished products **137** and reproduced the selenide catalyst (Scheme 59).

**Scheme 60:**
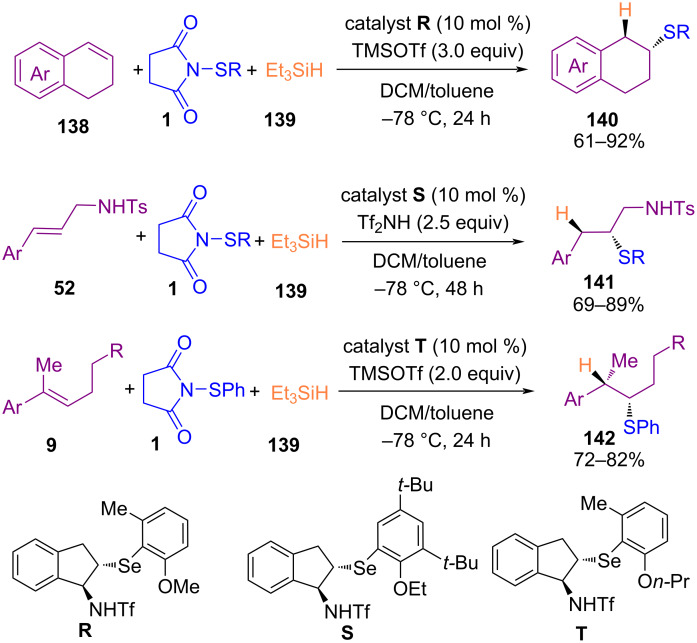
Chiral chalcogenide-catalyzed enantioselective hydrothiolation of alkenes.

**Scheme 61:**
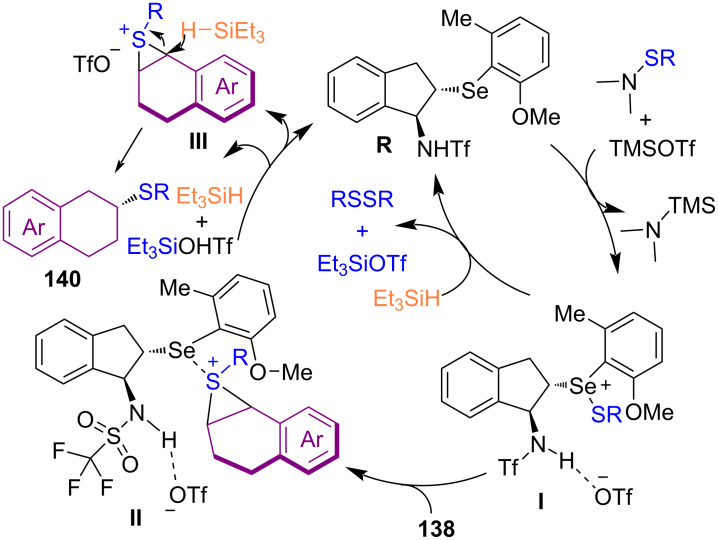
Proposed mechanism for chalcogenide-catalyzed enantioselective hydrothiolation of alkenes.

**Scheme 62:**
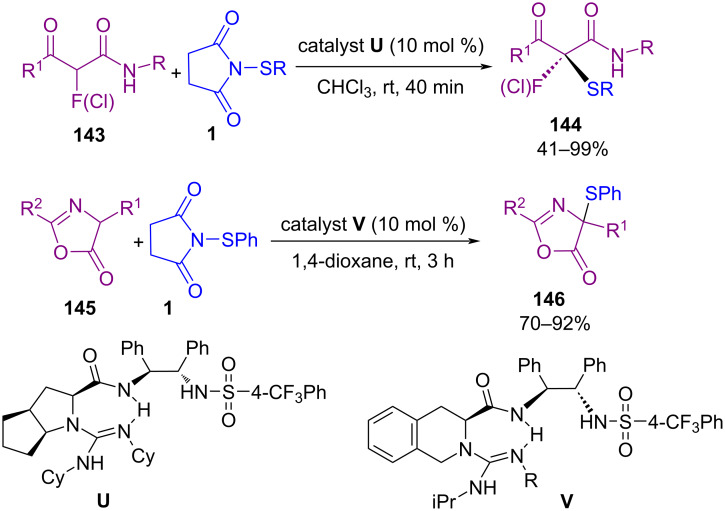
Organocatalytic sulfenylation for synthesis a diheteroatom-bearing tetrasubstituted carbon centre.

Zhao and co-workers found that *N*-thiosuccinimides are also suitable promoters for the enantioselective hydrothiolation of alkenes at low temperatures (Scheme 60) [91]. The synthesis of chiral sulfides up to 97% ee was achieved in this method. A wide range of cyclic alkenes **138** and acyclic alkenes **52** and **9** were smoothly tolerated in this organocatalysis strategy. According to the proposed mechanism, initially, the organocatalyst activated the electrophilic sulfur species to form intermediate **I** with the assistance of the Lewis acid. Intermediate **I** reduced by Et_3_SiH **139** to give thiol. Through the reaction of thiol with **I**, disulfide as a byproduct was formed, and intermediate **II** was generated by the reaction of **I** with **138**. Product **140** was obtained via direct hydride reduction of **II** by silane. On the other hand, most of **II** were converted to intermediate **III**, which underwent hydride reduction to render product **140** (Scheme 61). Another organocatalysis system was disclosed by Liu and co-workers for sulfenylation of α-fluoro-β-ketoamides **143** and azlactones **145** (Scheme 62) [92]. Besides α-fluoro-β-ketoamides, α-chloro-substituted ketoamide was also tolerated well in this transformation. Screening several chiral guanidines as the bifunctional catalyst revealed that these organocatalysts were suitable for the synthesis of α-fluoro/chloro-α-sulfenyl-β-ketoamides **144** and azlactone **146** skeletons. The presence of two heteroatom-bearing tetrasubstituted chiral carbon centers in a one-step fashion, avoiding the use of the heavy metal catalysts, and the performance of the reaction at ambient temperature are the prominent features of the protocol.

**Scheme 63:**
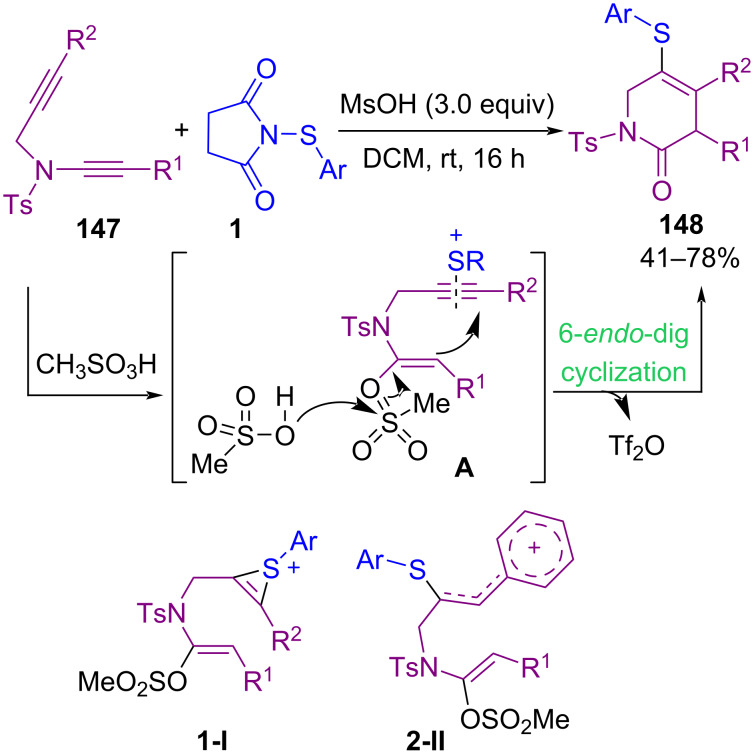
Thiolative cyclization of yne-ynamides.

Sahoo and co-workers found that sulfenylation of yne-tethered ynamide **147** with *N*-thiosuccinimides **1** was possible in the presence of only methanesulfonic acid in dichloromethane at room temperature (Scheme 63) [93]. The electrophilic activation of propargylalkyne **147** generated in situ a sulfonium cation **1-I**. Afterwards, 6-*endo*-dig cyclization of polarized ketene-*N*,*O*-acetal to the alkyne β-carbon and trapping of the sulfonium cation at the alkyne-α-carbon afforded 5-(arylthio)-3,6-dihydropyridin-2(1*H*)-one **148**. The coordination of a sulfonium electrophile to the C–C triple bond of **1-I** occurred through cyclopropyl intermediate **1-I**. The conversion of **1-I** to **2-II** was confirmed by mechanistic studies due to the stability of the benzyl carbocation, followed by 6-*endo*-dig cyclization. In this method, toxic transition metal catalysts, oxidants, or bases are not used, which made it economically and environmentally reliable.

**Scheme 64:**
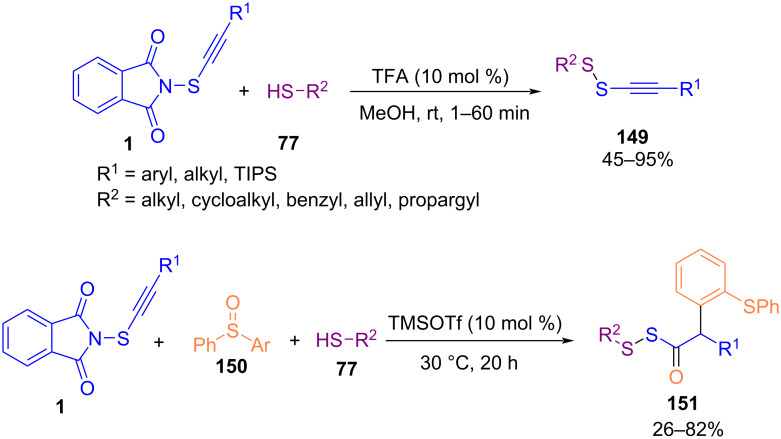
Synthesis of alkynyl and acyl disulfides from reaction of thiols with *N*-alkynylthio phthalimides.

In 2023, Gao et al. developed a metal-free procedure for the synthesis of functionalized alkynyl disulfides **149** and acyl disulfides **151** under acid catalysis (Scheme 64) [94]. In this regard, they used *N*-alkynylthiophthalimides in the reaction with thiols to make a series of bioactive disulfides. Various simple thiols, cystines, peptides, drugs and saccharides reacted smoothly with *N*-alkynylthiophthalimides in the presence of TFA as a catalyst. Also, aliphatic and aromatic thiols reacted with *N*-alkynylthiophthalimide and sulfoxide **149** to obtain acyl disulfides **151** through alkynylthiolation and hydrative oxyarylation in the presence of TMSOTf.

#### Catalyst-free sulfenylation by *N*-(sulfenyl)succinimides/phthalimides

**Scheme 65:**
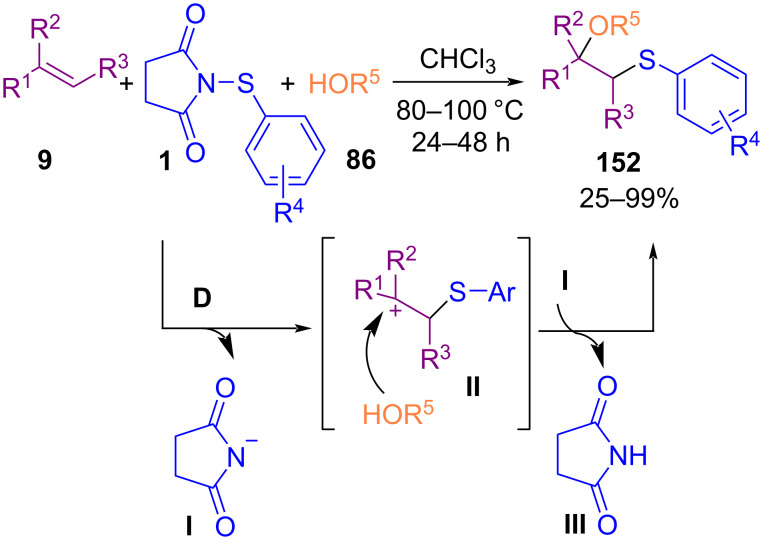
Oxysulfenylation of alkenes with 1-(arylthio)pyrrolidine-2,5-diones and alcohols.

**Scheme 66:**
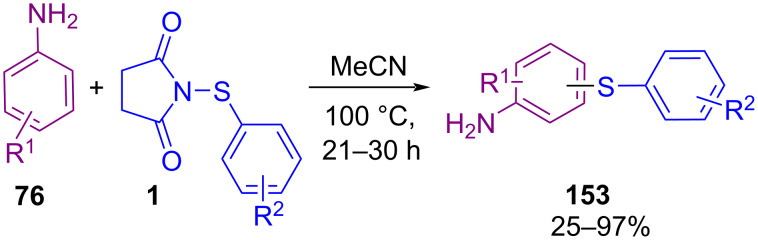
Arylthiolation of arylamines with (arylthio)-pyrrolidine-2,5-diones.

In 2015, oxysulfenylation of styrene derivatives **9** utilizing 1-(arylthio)pyrrolidine-2,5-diones **1** and alkyl/benzyl alcohols **86** toward β-alkoxy sulfides was developed by Fu et al. (Scheme 65) [95]. In this metal-free method, diverse β-alkoxy sulfides were synthesized without the need to any catalyst, or additive. The reaction proceeded through the formation of carbonium ion intermediate **I**, which underwent electrophilic addition of alcohol to provide product **152**. In the meantime, *N*-(arylthio)succinimide **1** as a thiolating reagent was used by another research team for the arylthiolation of arylamines **76** in acetonitrile as a solvent under metal-free conditions (Scheme 66) [96]. A broad spectrum of mono-, or diarylthiolated anilines **153** was obtained in low to excellent yields. Arylthiolation occurred predominantly at the *para*-position to the amino group, and when the *para*-position of aniline was occupied by another group, *ortho*-substituted products were identified.

**Scheme 67:**
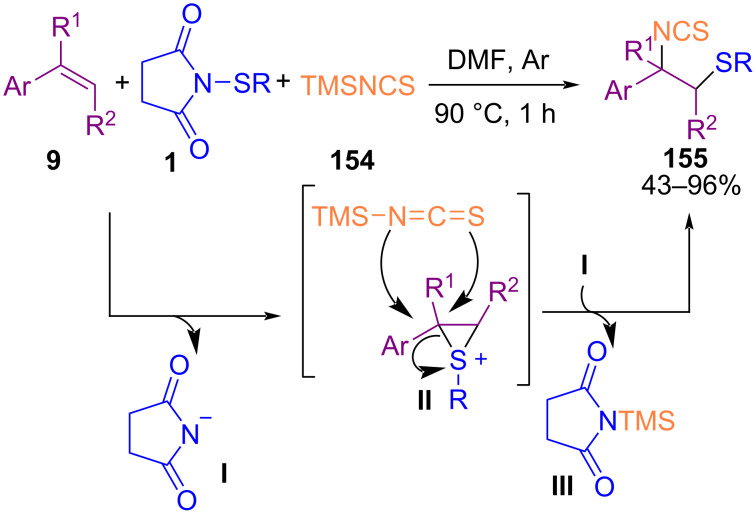
Catalyst-free isothiocyanatoalkylthiation of styrenes.

Fu′s research group established an isothiocyanatoalkylthiation of styrenes **9** in the presence of isothiocyanate **154** and *N*-(organothio)succinimides **1** under catalyst-free conditions (Scheme 67) [97]. The reaction proceeded through the formation of a three-membered cyclic intermediate **II** by the cleavage of **1** under thermal conditions. Between the nitrogen or sulfur atom in TMSNCS, electrophilic attack of nitrogen on **II** led to thermodynamically favored product **155**.

**Scheme 68:**
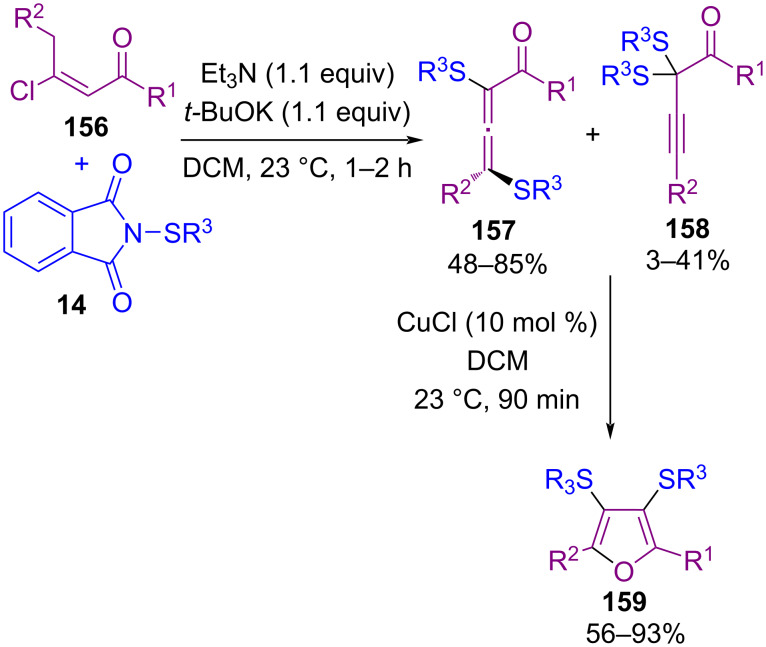
Sulfenylation of (*E*)-β-chlorovinyl ketones toward 3,4-dimercaptofurans.

In 2017, sulfenylation of (*E*)-β-chlorovinyl ketones **156** using *N*-(alkyl/arylthio)phthalimides **14** access to 3,4-dimercaptofuran skeletons **159** was presented by Kim and Oh et al. (Scheme 68) [98]. In the first step, by using Et_3_N and *t*-BuOK in the reaction of (*E*)-β-chlorovinyl ketones **156** and *N*-(phenylthio)phthalimide **14**, a series of α,γ-dithioallenyl ketones **157** and α,α-dithiopropargyl ketones **158** were obtained in a different ratio. In the second phase, by adding copper chloride as a catalyst in the reaction medium, 3,4-dimercaptofurans **159** were formed via 1,2-sulfur migration.

**Scheme 69:**
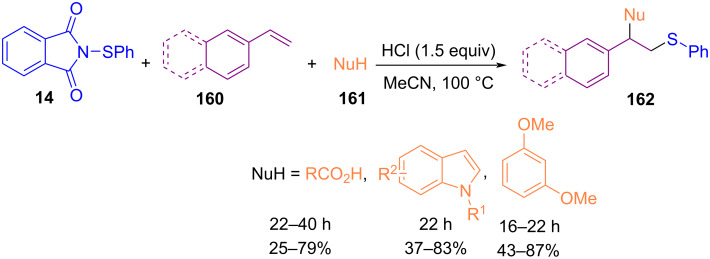
HCl-promoted intermolecular 1, 2-thiofunctionalization of aromatic alkenes.

**Scheme 70:**
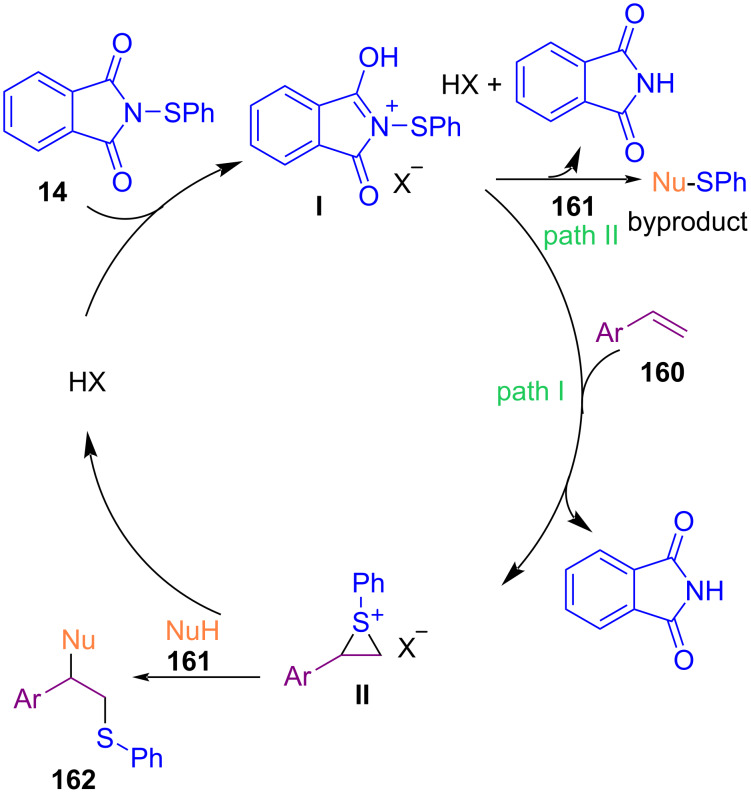
Possible mechanism for HCl-promoted 1,2-thiofunctionalization of aromatic alkenes.

In 2018, Shen′s research team disclosed a new protocol for 1,2-thiofunctionalization of arylalkenes **160** with *N*-arylthiophthalimide **14** and various nucleophiles, including aryl ethers, carboxylic acids, indoles, and pyrroles in the presence of HCl (Scheme 69) [99]. The procedure utilized no toxic metal catalyst, or additive, which made it economically and environmentally reliable. According to the mechanism, two pathways occurred after the formation of intermediate **I** by the reaction of **14** with HX. In path I, intermediate **I** reacted with alkene **160** to give intermediate **II**, which underwent a nucleophilic attack of **161** to give the product **162** and regenerated HX. In path II, **I** reacted with nucleophile **161** to produce a byproduct, phthalimide, and HX (Scheme 70). The coupling reaction was influenced by nucleophilic properties and the steric effect of the nucleophile reagents. In the same year, the treatment of amines with *N*-thiophthalimides led to sulfenamides promoted by 2-ethoxyethanol under microwave irradiation [100]. Alkylamines, such as morpholine, cyclohexylamine, pyrrolidine, and *tert*-butylamine were participated in this coupling process. All reactions occurred in a shorter time with higher chemical yields compared to the traditional heating methods.

**Scheme 71:**
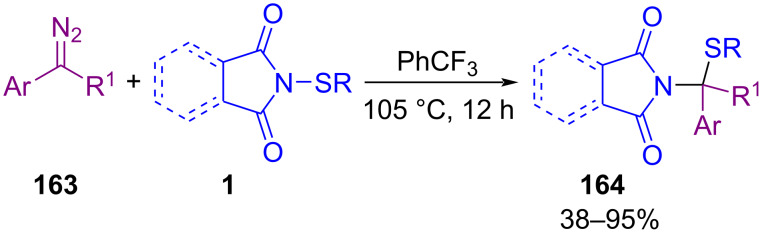
Coupling reaction of diazo compounds with *N*-sulfenylsuccinimides.

In the meanwhile, Qiu and Xu et al. reported the coupling reaction between diazo compounds **163** and *N*-sulfenylsuccinimides **1** under catalyst-, base-, and additive-free conditions (Scheme 71) [101]. The reaction proceeded via a radical pathway, in which a free carbene was generated under heating, followed by the formation of ylide, N–S bond cleavage, and C–N bond formation along with the release of N_2_.

**Scheme 72:**
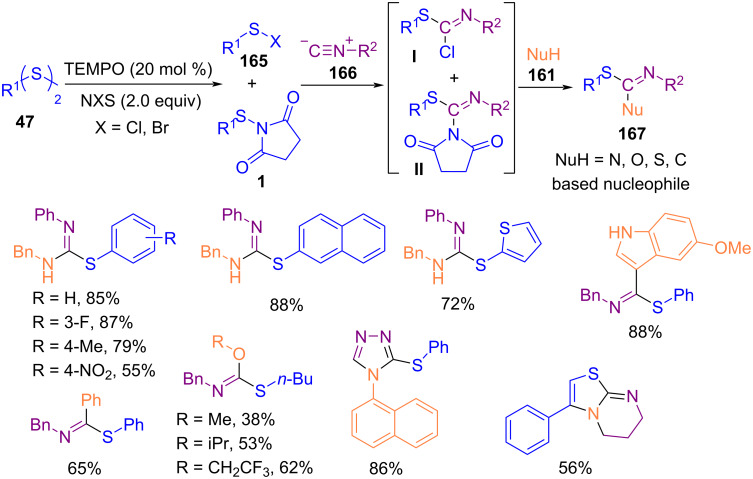
Multicomponent reactions of disulfides with isocyanides and other nucleophiles.

In 2019, Sun and co-workers introduced an unprecedented method for the synthesis of isothiourea derivatives via the activation of diaryl/alkyl disulfides **47** with *N*-halosuccinimides in the presence of TEMPO, followed by insertion of an isocyanide molecule **166** or other nucleophiles **161** (Scheme 72) [102]. By studying the spectroscopic evidence, the authors found that both sulfenyl halide **165** and *N*-sulfenylsuccinimide **1** intermediates were involved in the reaction. Removal of TEMPO as a radical initiator from the reaction mixture did not result in product formation so, it seems that the reaction moved through a radical route for the formation of sulfenyl halide **I**, and *N*-sulfenylsuccinimide **II**. The use of azobisisobutyronitrile (AIBN) instead of TEMPO also resulted in 85% yield of the product, while benzoyl peroxide (BPO) gave a low yield. Various nucleophiles **161**, including ammonia, alkylamines, hydrazines, alcohols and alkoxides, indole, *N*-alkylpyrrole, *N*-substituted anilines, PhSH, and PhMgBr worked well under these conditions. Asymmetric thiolation of 4-substituted pyrazolone derivatives with *N*-thiophthalimides catalyzed by 1 mol % of chiral iminophosphorane organocatalyst was carried out under mild conditions [103]. Solvent control in the procedure can affect the yield of products due to the solubility of the catalysts. Various solvents, such as acetone, ethyl acetate, tetrahydrofuran, methanol, toluene, hexane, and *n*-pentane were employed, in which the products in non-polar hydrocarbon solvents like hexane and *n*-pentane were obtained in excellent efficiency and enantioselectivity.

**Scheme 73:**
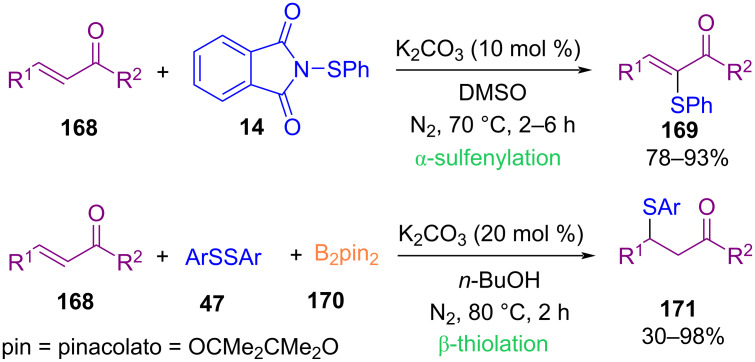
α-Sulfenylation and β-sulfenylation of α,β-unsaturated carbonyl compounds.

Song et al. found that the chemoselective α-sulfenylation and β-thiolation of α,β-unsaturated carbonyl compounds **168** can be achieved with *N*-thiophthalimides **14** and diaryl disulfides **47**, respectively (Scheme 73) [104]. They remarked that the presence of B_2_pin_2_ was essential in the coupling reaction of disulfides with α,β-unsaturated carbonyl compounds **168**. The sulfenylation involved a 1,4-addition of the phthalimide anion to the β-carbon of chalcone, followed by electrophilic sulfur attack and deprotonation. In the thiolation, in situ formation of thiophenol occurred, followed by thio-Michael addition of chalcone with thiophenol. *N*-Calcogenophthalimide also can be used to prepare thiophosphates, thiophosphinates and selenophosphates by reaction with the P(O)H moieties of H-phosphonates [105].

## Conclusion

To date, both metal-catalyzed and organocatalyzed C–S bond formations have been widely expanded. In particular, organocatalytic methodologies are effective for direct construction of stereogenic carbon centers bearing a sulfur atom. Although, significant efforts have been made to form enantioselective C–S bonds, the direct sulfenylation with more green, economical, and environmentally friendly sulfenylating reagents remains a challenge for organic chemists. *N*-(Sulfenyl)succinimides/phthalimides as new alternative sulfenylating reagents can meet this demand. In this context, we observed that most of the reactions have used unactivated C–H bonds, such as C(sp^2^)–H and C(sp^3^)–H bonds. In some reactions, chiral organocatalysts catalyzed asymmetric sulfenylation processes. In most cases, there is no need to use a metal catalyst, base, or additive. *N*-(Sulfenyl)succinimide/phthalimide acted as an active electrophilic sulfur source, acted in the reaction mechanisms. However, mechanistic studies need further exploration to define a valid reaction pathway. Therefore, we believe that the use of *N*-(sulfenyl)succinimide/phthalimide in chemical syntheses will be widely seen in the future.
